# A Major Locus on Wheat Chromosome 7B Associated With Late-Maturity α-Amylase Encodes a Putative *ent*-Copalyl Diphosphate Synthase

**DOI:** 10.3389/fpls.2021.637685

**Published:** 2021-02-26

**Authors:** Adinda Derkx, Ute Baumann, Judy Cheong, Kolumbina Mrva, Niharika Sharma, Margaret Pallotta, Daryl Mares

**Affiliations:** ^1^School of Agriculture, Food and Wine, University of Adelaide, Glen Osmond, SA, Australia; ^2^South Australian Agricultural Research Institute, Waite Precinct, Glen Osmond, SA, Australia; ^3^NSW Department of Primary Industries, DPI Research and Business Excellence, Orange, NSW, Australia

**Keywords:** quantitative trait locus, LMA, gibberellins, seed development, *Triticum aestivum* L., sequence capture

## Abstract

Many wheat varieties have the potential to develop unacceptably high levels of α-amylase in the grains if exposed to a cool temperature shock or simply cool temperature during the early to middle stages of grain filling. This phenomenon is referred to as late maturity α-amylase (LMA). The enzyme persists in the grain until harvest and may result in wheat with a low Falling Number that does not meet receival and export specifications. Resistance to LMA is therefore a valuable target for wheat breeders and wheat industries in general. Genetic evidence implicating a locus on the long arm of chromosome 7B in variation in LMA phenotype was confirmed in this investigation. Through intensive fine-mapping an *ent*-copalyl diphosphate synthase (CPS), hitherto named *LMA-1*, was identified as the likely candidate gene associated with variation in LMA phenotype. Single Nucleotide Polymorphisms (SNPs) within the *LMA-1* coding sequence of Chinese Spring, Maringa and Halberd result in either prematurely terminated or functionally altered proteins that are associated with useful levels of resistance to LMA. *LMA-1* transcripts detected in de-embryonated grain tissue from around 15 days after anthesis, several days before the synthesis of α-amylase, were low in the resistant varieties Chinese Spring and Maringa compared with LMA susceptible genotype Spica. This was associated with a dramatic reduction in the concentrations of intermediates in the gibberellin biosynthesis pathway such as GA_19_, evidence that *LMA-1* was functioning as CPS in the gibberellin biosynthesis pathway. A survey of a large collection of Australian and international wheat varieties distinguished 9 major haplotypes at the *LMA-1* locus. Generally, within classes, there was notable variation for LMA phenotype and evidence for genotypes whose resistance is presumed to be due to genetic loci located elsewhere on the wheat genome. Further investigation is required to characterize the sequence of steps between *LMA-1* and α-amylase synthesis as well as to gain a better understanding of the role and potential impact of other genetic loci. Diagnostic markers for sources of resistance and SNP variation reported in this study should assist breeders to deploy resistance associated with *LMA-1* variants in breeding programs.

## Introduction

Late-maturity α-amylase (LMA) in wheat involves the premature synthesis of high pI α-amylase, encoded by homologous *α-Amy-1* genes located on the long arms of the group 6 chromosomes. This may occur during grain development in some varieties depending on the environmental conditions (Mares and Mrva, 2008). The α-amylase protein, once synthesized, is retained in the grain at harvest-ripeness and may cause the grain to be unacceptable for receival into milling grades and export because of the low Falling Number. Production of high levels of α-amylase can also occur during pre-harvest sprouting, when germination of ripe grain is initiated by rain prior to harvest (Mares and Mrva, 2014). The α-amylase that is synthesized during the early stages of sprouting is the same high pI α-amylase found in LMA-affected grain and differentiation between the two phenomena in the harvested grain is very difficult. Marker-assisted breeding for both traits would go a long way toward reducing their impacts on growers, millers and bakers, and while there has been good progress on pre-harvest sprouting resistance, there has by comparison been relatively little progress on LMA. Due to the complex interaction between genotype, temperature and stage of grain development underlying LMA expression (Mares and Mrva, 2014; Derkx and Mares, 2020), the incidence and severity of LMA are very difficult to predict and as a consequence, it poses a significant challenge to growers, wheat breeders and the wheat industry as a whole. Currently, the management of this trait, at least in Australia, is based on phenotyping varieties before release to growers using conditions that maximize the expression of LMA (Mrva and Mares, 2001a; Derkx and Mares, 2020). Unfortunately, this is both labor intensive and expensive and in practice is limited to the final stages of a breeding program. Selection at this late stage of the breeding program involves a waste of resources used during the development of the culled lines and reduced opportunity to realize genetic advances in other important traits. A better understanding of the genetic and environmental control mechanisms and the development of molecular tools for selection early in the breeding program would be of considerable benefit to wheat breeders. Mrva and Mares (2001b) reported the first QTL to be associated with variation in LMA phenotype in a Cranbrook (LMA-susceptible) x Halberd (LMA-resistant) doubled haploid population; a highly significant locus on the long arm of chromosome 7B and a minor locus on 3B. Over the intervening period since that report, a large number of populations involving different sources of LMA-susceptibility (from here on referred to as LMA-sus) and LMA-resistance (LMA-res) have been investigated with the aim of confirming the role of the 7BL locus and identifying additional genetic loci. Neither the identity of the 7BL gene nor the pathway that links the gene to the initiation of the coordinated transcription of the *α-Amy-1* genes located on the long arms of the group 6 chromosomes has been reported. A starting point for fine-mapping the 7BL locus was a comparison of the published QTL maps for LMA (Mrva and Mares, 2001b) and boron tolerance, *Bo1* (Jefferies et al., 2000; Schnurbusch et al., 2007) in the population Cranbrook (LMA-sus, boron susceptible)/Halberd (LMA-res, boron tolerant). In both maps, the QTL appeared to be located in the same region of 7BL near the marker psr680. Subsequent phenotyping and marker testing failed to identify any recombinants with LMA-sus and boron tolerance or the alternate combination and it was concluded that the two loci were closely linked (Mares and Mrva, 2008).

Temperature during grain development is associated with variation in the expression of LMA (Lunn et al., 2001; Farrell and Kettlewell, 2008; Mrva et al., 2008; Mares and Mrva, 2014; Derkx and Mares, 2020). Tall LMA genotypes, and semi-dwarfs with reduced plant height that is not due to gibberellin insensitivity, express LMA under a wide range of temperatures. By contrast, for gibberellin–insensitive semi-dwarfs, LMA expression is reduced or inhibited when daily maximum temperatures rise above 20–25°C. For gibberellin-insensitive semi-dwarfs ripening under warmer temperatures, LMA can be induced by the application of a cool temperature shock of several days duration (Mrva and Mares, 2001a; Derkx and Mares, 2020). This response to a cool temperature shock has formed the basis of a routine screening protocol that is used in Australia to reduce the risk of LMA-sus genotypes being passed on to wheat growers.

While the biochemical mechanisms involved in LMA have not been defined, the parallel with α-amylase synthesis in germination has focused attention on the gibberellins. The observed reduction in LMA in semi-dwarf wheat varieties based on *Rht-B1b*, *Rht-D1b*, and *Rht-B1c* that are associated with insensitivity to gibberellin (Mrva and Mares, 1996), but not varieties based on alternate semi-dwarfing genes such as *Rht8* (Mrva et al., 2008), would appear to support a similar role for this hormone in LMA. Elevated gibberellin levels at mid-grain development have been reported to be associated with LMA (Barrero et al., 2013; Kondhare et al., 2014) while Kondhare et al. (2015) suggested that the sensitivity of grains to, or levels of, ABA and gibberellin could be involved. Barrero et al. (2013) also demonstrated that there were substantial differences in gene expression profiles during the early stages of grain development between LMA-sus and LMA-res genotypes.

This research had the following aims: The first was to identify the gene underlying the LMA QTL on wheat chromosome 7B. Secondly to develop molecular markers to aid the selection and breeding of wheat with lowered LMA risk. Finally, to gain a better understanding of the likely pathway that links the gene with the transcription of the *α-Amy-1* genes and the synthesis of high pI α-amylase in the aleurone of developing wheat grain. The broader applicability of the results obtained in this study was then assessed by surveying a panel of cultivated varieties for 7B genotype and LMA phenotype when grown in three different environments.

## Materials and Methods

### Germplasm

Wheat varieties and populations were grown in the field or glasshouses at the Waite Campus of The University of Adelaide, Australia. Pedigrees, country of origin, *Rht* genotype, and LMA phenotype of main varieties used in this study are listed in Supplementary Table 1. Seed of Australian varieties was obtained from the respective breeding companies or from stocks maintained at the University of Adelaide while overseas varieties were sourced from the Australian Grains Genebank, Horsham, Victoria, Australia. Cranbrook (LMA-sus)/Halberd (LMA-res) 7BL recombinants were provided by Schnurbusch et al. (2007). Spica (LMA-sus)/Maringa (LMA-res) doubled haploid lines Spica/Maringa52 (LMA-sus) and Spica/Maringa47 (LMA-res) were sourced from stocks held at the University of Adelaide and previously described in Barrero et al. (2013). Nainari *rht/Rht-D1b* isolines and Maringa *rht* came from the Australian Grain Genebank whilst Maringa *Rht-D1b* was kindly provided by Dr. Peter Chandler (CSIRO).

### Populations for QTL Discovery and Fine-Mapping

Doubled haploid (DH) and single seed descent from F_2_ (SSD) populations were derived from crosses between the LMA-res lines, Halberd, Maringa and Chinese Spring, and ten LMA-sus lines (Table 1). DH populations were developed using the wheat × maize (*Zea mays*) system (Kammholz et al., 2001). SSD populations were taken to F_6_. Where one parent was a semi-dwarf, sequential selection for tall plants was applied from F_2_ onwards to overcome the confounding effects of semi-dwarfing genes, *Rht-B1b* and *Rht-D1b*, and simplify phenotyping.

**TABLE 1:** Populations derived from crosses between LMA-res and LMA-sus varieties.

LMA resistant line	LMA susceptible line	Population structure	Number of lines
Halberd*	**Kennedy**	Doubled haploid	161
Halberd*	**Seri 82**	Doubled haploid	181
Halberd	RAC655*	SSD, F_6_	227
Halberd	BD159*	SSD, F_6_	248
Halberd	Chara*	SSD, F_6_	200
Halberd	Reeves*	SSD, F_6_	151
Halberd*	**Wyalkatchem**	SSD, F_6_	171
Halberd*	**ChuanMai 18**	SSD, F_6_	143
Halberd*	AUS33402	SSD, F_6_	160
Maringa	Spica*	Doubled haploid	173
Chinese Spring*	Spica	SSD, F_6_	140

Semi-dwarf lines in bold. *Female parent.

Fine-mapping was performed by using recombinants from large Chinese Spring (LMA-res)/Spica (LMA-sus) and Spica (LMA-sus)/Maringa (LMA-sus) F_2_ grain populations (>5000 lines) derived from reciprocal crosses between the named parents and seed harvested from F_1_ plants.

### Plant Growth Conditions

Plants were grown in a standard glasshouse (glasshouse 1) equipped with evaporative air-conditioning but where temperature varied due to differences in daily solar radiation. Where plants were to be given a cool temperature shock, sowing time was adjusted such that grain ripening occurred when daily maximum temperatures were likely to be between 25 and 30°C. Alternatively, plants were grown in a glasshouse equipped with heating, cooling and supplementary lighting (E602G 630 W Grow Lights, Heliospectra, Göteborg, Sweden). Temperatures were set at 22–23°C day and 15°C night on a diurnal cycle with a photoperiod of 14 h as described in Derkx and Mares (2020) (glasshouse 2).

### Phenotyping of a Wheat Panel

A panel of 212 wheat varieties was screened in a replicated and randomized experiment, using the cool to be consistent shock method, for LMA phenotype in glasshouse 1 in the winter of 2018. Mean minimum and maximum temperatures for the period between anthesis and cool temperature treatment were 11 and 23°C, respectively, whereas the minimum and maximum temperatures recorded were 6.8 and 28.5°C. A subset comprising 153 varieties was repeated in glasshouse 1 in the summer of 2018/19, again using the cool shock method. For this experiment, mean minimum and maximum temperatures for the period between anthesis and cool temperature treatment were 15.5 and 27.5°C, respectively, whereas the minimum and maximum temperatures recorded were 10.5 and 30.5°C. A further subset comprising 76 varieties was grown, in two replicated and randomized experiments, under constant lower temperature conditions, without cool shock treatment, in the winter of 2019 in glasshouse 2. The collection comprised a large number of Australian lines that included nearly all varieties released in the decade up to 2017, as well as the majority of varieties that were examined by sequence capture, and a selection of Canadian and European varieties obtained from the Australian Grains Genebank (Horsham, VIC, Australia). Plants were grown in 9 cm square 17 cm deep pots with two plants per pot and two replicate pots per line. Four tillers per pot were tagged at anthesis (first anthers extruded; Zadoks growth stage 61) and sampled at 30 (winter) or 28 (summer) days post-anthesis for cool-shock induction of LMA, which was performed as described in Derkx and Mares (2020). The grain from the 4 spikes per pot was pooled into a single sample for subsequent estimation of high pI α-amylase content by ELISA.

### Phenotyping of Mapping Populations

The populations Chinese Spring (LMA-res)/Spica (LMA-sus) and Spica (LMA-sus)/Maringa (LMA-res), and the F_2_-derived recombinants selected from the F_2_ grain populations were grown to ripeness in 9 cm square 17 cm deep pots in glasshouse 1 and 2, respectively. Embryo ends from 5 harvested grains were removed and endosperm portions were pooled and milled to a fine meal and the amount of high pI α-amylase protein determined. These varieties were all tall (*Rht-B1a, RhtD1a*) genotypes and expression of LMA in the glasshouse was constitutive and did not require a cool temperature shock (Mares and Mrva, 2008).

Semi-dwarf recombinants from Cranbrook (LMA-sus)/Halberd (LMA-res) (Schnurbusch et al., 2007) and varieties that required a cool temperature shock treatment were grown in glasshouse 1 and phenotyped using a modification of the protocol reported by Mrva and Mares (2001a). Instead of total α-amylase activity, the level of LMA was determined using the high pI α-amylase-specific ELISA (Verity et al., 1999) to measure the amount of α-amylase protein, reported here as optical density (OD) units. This modification eliminated potential confounding effects due to any residual low pI α-amylase retained from early grain development.

### Chemicals and Antibodies

Polyclonal and monoclonal antibodies required for LMA ELISA were covered by a sub-license to the University of Adelaide by the Grains Research and Development Corporation, Australia, and were provided from stocks maintained by the South Australian Research and Development Institute, Urrbrae, South Australia. Labeled (horseradish peroxidase, HRP) anti-mouse antibody was sourced from Merck Australia, while the substrate for HRP, Blue Substrate ESBP1000, was obtained from ELISA Systems, Queensland, Australia. Other chemicals used in the study were obtained from Sigma-Aldrich Australia.

### Determination of High pI α-Amylase Content

De-embryonated grain was milled into a fine meal using a Perten LM-3310 laboratory burr mill equipped with type-2 fine grinding disks (Perten Instruments, Macquarie Park, NSW, Australia). Four 100 mg technical replicates per grain sample were assessed for high pI α-amylase content using a sandwich ELISA assay developed by Verity et al. (1999) using the protocol described in Barrero et al. (2013) adapted to a 96-well format. Spectrometric measurements were performed using a Benchmark Plus microplate reader (Bio-Rad Laboratories, Hercules, CA, United States) at 595 nm, and results were expressed in optical density (OD).

### Extraction and Quantification of Gibberellins

Developing grain on floret positions 1 and 2 of seven spikes per sample was collected at 26 days post-anthesis, frozen in liquid nitrogen, freeze-dried and milled into a fine meal with a RotoMix capsule mixing unit (3M ESPE, St. Paul, MN, United States) for 7 s using a 4 mm stainless steel ball-bearing. Gibberellins were extracted and quantified using UPLC/ESI-MS/MS at the Aquatic and Crop Resource Development Research Centre of the National Research Council Canada^1^. An aliquot (100 μL) containing all the internal standards, each at a concentration of 0.2 pg μL^–1^, was added to homogenized sample (50 mg freeze-dried, ground wheat grain). 3 mL of isopropanol: water:glacial acetic acid (80:19:1, v/v/v) were further added, and the samples were agitated in the dark for 24 h at 4°C. Samples were centrifuged and the supernatant dried on a Büchi Syncore Polyvap (Büchi, Switzerland). Further, they were reconstituted in 100 μL acidified methanol, adjusted to 1 mL with acidified water, and then partitioned against 2 mL hexane. After 30 min, the aqueous layer was isolated and dried as above. Dry samples were reconstituted in 800 μL acidified methanol and adjusted to 1 mL with acidified water. The reconstituted samples were passed through equilibrated Sep-Pak C18 cartridges (Waters, Mississauga, ON, Canada), the eluate being dried on a LABCONCO centrivap concentrator (Labconco Corporation, Kansas City, MO, United States). An internal standard blank was prepared with 100 μL of the deuterated internal standards mixture. A quality control standard (QC) was prepared by adding 100 μL of a mixture containing all the analytes of interest, each at a concentration of 0.2 pg μL^–1^, to 100 μL of the internal standard mix. Finally, samples, blanks, and QCs were reconstituted in a solution of 40% methanol (v/v), containing 0.5% acetic acid and 0.1 pg μL^–1^ of each of the recovery standards. Hormones were quantified by HPLC-ESI-MS/MS as described in detail by Chiwocha et al. (2003, 2005). Samples were injected onto an ACQUITY UPLC HSS C18 SB column (2.1 mm × 100 mm, 1.8 μm) with an in-line filter and separated by a gradient elution of water containing 0.02% formic acid against an increasing percentage of a mixture of acetonitrile and methanol (50:50, v/v). The analysis utilized the Multiple Reaction Monitoring (MRM) function of the MassLynx v4.1 (Waters Inc) control software. The resulting chromatographic traces were quantified off-line by the QuanLynx v4.1 software (Waters Inc) wherein each trace was integrated and the resulting ratio of signals (non-deuterated/internal standard) compared with a previously constructed calibration curve to yield the amount of analyte present (ng per sample). Calibration curves were generated from the MRM signals obtained from standard solutions based on the ratio of the chromatographic peak area for each analyte to that of the corresponding internal standard, as described by Ross et al. (2004). The QC samples, internal standard blanks and solvent blanks were also prepared and analyzed along each batch of tissue samples.

### DNA Isolation

DNA was extracted from young leaf tissue using a standard phenol/chloroform method (Rogowsky et al., 1991; Pallotta et al., 2000) for gene sequencing. For KASP assays^2^, DNA was extracted from freeze-dried tissue using a standard 96-well plate method (Pallotta et al., 2003), quantified using a NanoDrop 1000 spectrophotometer (Thermo Fischer Scientific, Wilmington, DE, United States) and diluted to 30 ng μl^–1^. An additional step was incorporated for 96-well plate DNA extraction from embryo sections of mature grain; sections were placed separately in 1.1 ml microcentrifuge tubes and imbibed in 50 μl Milli-Q water for 24 h at room temperature in the dark, then frozen at −80°C for a minimum of 3 h before freeze-drying.

### Genotyping, QTL Discovery and Marker Development

Map Manager QTXb19 (Manly et al., 2001) was used to perform linkage and interval analyses. Microsatellite markers on chromosomes 7B (gwm577, wmc276, wmc273, gwm146, wmc276, and barc182) from Röder et al. (1998) and Somers et al. (2004), were selected based on the chromosomal location of a previously published LMA QTL and tested for possible linkage with LMA in the Spica/Maringa DH population and Chinese Spring/Spica SSD population.

Genomic sequences of Chinese Spring chromosome 7B that we had access to during the duration of the project were submitted to the TriAnnot pipeline (Leroy et al., 2012) for identification of putative gene coding regions. Initially, targeted 7BL-derived BAC sequences from preliminary genome assemblies were kindly provided by Prof. Odd-Arne Olsen and we used the genic sequences for marker design (CAT, PPK, CRF, SPA, UN2, sc111, SOS1, HMG, CPS2, CPS1, MATE1, MYB, HYPB, coGST, and MATE2). A second approach was based on Illumina HiSeq read alignment of the Bioplatforms Australia (BPA) accessions Alsen, Baxter, Drysdale, Gladius, Kukri, RAC875, and Wyalkatchem (Edwards et al., 2012) to the scaffolds (Ref v0.4) released by the International Wheat Genome Sequencing Consortium (IWGSC). After alignment, the resulting bam files were further processed using SAMtools version 1.2 (Li et al., 2009) commands for sorting, indexing and creation of an mpileup output for variant identification. An in-house python script was used for SNP identification between the Chinese Spring reference and the named BPA accessions for the candidate region. Identified SNPs were targeted for the development of Kompetitive Allele-Specific PCR (KASP) markers (Supplementary Table 2) and assayed using KASP Mastermix and a SNPline system from LGC Genomics.

### Sanger Sequencing

Sanger sequencing of parts of the Maringa and the complete Spica *LMA-1* gene was performed by AGRF (Australian Genome Research Facility, Australia) on PCR products generated using Immolase DNA polymerase [Bioline (Aust) Pty. Ltd., Alexandria, NSW, Australia] and purified using ISOLATE II PCR and Gel Kit (Bioline).

### Sequence Capture and NGS Sequencing

The sequence of a 23 kb candidate region was sent to Arbor Biosciences (Ann Arbor, MI, United States) for the design of capture probes together with DNA from selected recombinants and genotypes representing our observed range in LMA phenotypes. Genomic DNA samples were sent fully dried down in 1.5 mL tubes to Arbor Biosciences for targeted sequencing as part of a myReads NGS service project.

There, samples were resuspended in 105 μL nuclease-free water and then quantified using a QuantiFluor dsDNA system (Promega). A portion of each sample (962 to 4000 ng) was then sonicated in a Q800R sonicator (QSonica) and then size-selected with SPRI beads to mean fragment lengths of roughly 300 bp, yielding between 44 and 628 ng final material. Portions of this (between 35 and 500 ng) were taken to TruSeq-style Illumina NGS library prep, which included six cycles of amplification using dual 8 bp index primers using KAPA Hot-start HiFi polymerase system. This yielded libraries totaling 1111 to 2231 ng each.

For target enrichment, 200 ng of each of four libraries were combined equimolar into capture pools (800 ng total library mass per pool). These were then enriched using the myBaits system version 3 with a custom probe set comprised of 861 individual 80 nucleotide probes meant to capture roughly 23 kb of the wheat genome. Hybridization and wash conditions were all according to the standard version 3 procedure, though Block 1 (human C0T-1 DNA) was replaced with an equal volume of Developer Reagent (Roche). After washing, half of the resulting bead-bound enriched library (15 μL) was taken to 11 cycles of amplification with KAPA HiFi using the universal P7/P5 primer pair, purified with SPRI beads and then quantified with library quantitative PCR. The ten individual capture pools were then combined equimolar and submitted for sequencing on an Illumina HiSeq 2500 instrument using a paired-end 125 bp sequencing protocol. After standard CASAVA post-processing, the reads were de-multiplexed using both 8 bp indexes and delivered by Arbor Biosciences as Read 1 and Read 2 FASTQs. For pre-processing of raw reads, read qualities were inspected with FASTQC version 0.11.4^3^ before and after quality and adapter trimming. Trimming was achieved with Trimmomatic version 0.36 (Bolger et al., 2014), using the following parameters: -phred33 LEADING:5 TRAILING:5 SLIDINGWINDOW:4:20 MINLEN:50. For the sequence capture data clean reads were aligned to the CS IWGSC RefSeq v0.4 and v1.0 by Bowtie2 version 2.3.0 (Langmead and Salzberg, 2012) allowing a 2% mismatch rate with the following parameters: –end-to-end –very-sensitive –n-ceil L,0,0.1 –rdg 3,3 –rfg 3,3 –no-unal –mp 6,6 –np 4 –no-mixed –score-min L,0,−0.12. Since at the time, most available genome browsers did not support the CSI indexing schema every pseudomolecule was split into parts as described in Watson-Haigh et al. (2018). Whole genome shotgun Illumina reads from the BPA accessions were aligned to RefSeq v0.4 as described in Watson-Haigh et al. (2018). For visualization of aligned reads, we imported the sorted and indexed bam files into Integrated Genome Browser (IGV) (Robinson et al., 2011).

### Protein Modeling

The protein structure of LMA-1 from Spica was modeled, at high confidence, using the Phyre^2^ server^4^ (Kelley et al., 2015) and three-dimensional structural data from the *Arabidopsis thaliana ent-*copalyl diphosphate synthase crystal structures available in the Protein Data Bank (3pyb, chain B). Amino acid substitutions were investigated with the web-based Missense3D software (Ittisoponpisan et al., 2019). Images were generated with the PyMOL Molecular Graphics System, Version 1.2r3pre, Schrödinger, LLC^5^.

### *In silico* Prediction of SNP Effects

Several prediction algorithms were employed to evaluate the effect of non-synonymous amino acid substitutions in the LMA-1 protein. These included: PredictSNP (Bendl et al., 2014), a consensus classifier that generates a prediction from the output of several prediction tools, SNAP^2^ (Hecht et al., 2015), Provean (Choi et al., 2012), and also the evaluations from Phyre^2^ and Missense3D.

### RNA Isolation and Gene Expression

Transcript abundance of *LMA-1* was determined using three biological replicates consisting of 20 seeds collected at some or all of the stages 10, 15, 18, 21, 24, 27, and 30 days post-anthesis (DPA) from plants grown in glasshouse 2. After removing the embryo section, the de-embryonated seeds were crushed in liquid nitrogen using mortar and pestle. 100 mg of the crushed frozen tissue was transferred into cold Eppendorf tubes that were immediately transferred to liquid nitrogen then stored at −80°C for RNA extraction. Total RNA was isolated using Spectrum Plant Total RNA kit (Sigma) with an on-column DNase treatment. The concentration and integrity of the RNA were determined using a NanoDrop 8000 (Thermo Fischer Scientific). RNA samples with A260/A280 ratio above 1.8 and A230/A260 ratio above 2.0 were used for cDNA synthesis. SuperScript III Reverse Transcriptase kit (Life Technologies) was used to synthesize the cDNA. The reaction consisted of a 500 ng aliquot of purified RNA from each sample in a final reaction volume of 20 μl, performed according to the manufacturer’s instructions. The cDNA was diluted 5 times in sterile Milli-Q water before being utilized as a template in the qRT-PCR reactions.

Primers were designed such that the forward primer spanned an intron and the reverse primer was specific to the Chinese Spring 7B homoeologue (CPS2qPCR_F: GATGTT ACTTTCTGAGGAC; CPS2qPCR_R: TCTTTCACTAGCACTC CTTAC). For the amplification of the gene in *Bo-1* cultivars, the use of a different primer set was required due to allelic divergence (Yitpi_F: CCTCCAGGAGCACGGCGACG; Yitpi_R: TTCCTT CCACTAGCACTCCT). Specific amplification for the gene was confirmed by a single, distinct peak in melt curve analysis and also ensuring the qRT-PCR product fragment sizes were correct (345 and 315 bp, respectively). qRT-PCR products were sequenced to verify that the amplification was correct.

The qRT-PCR assays were prepared according to the manufacturer’s instructions using SsoAdvanced Universal SYBR Green Supermix (Bio-Rad Laboratories, Inc.). Amplifications were performed in a CFX384 Touch Real-Time PCR Detection System (Bio-Rad Laboratories, Inc.) with 3 min of 95°C followed by 40 cycles of 30 s at 55°C, and fluorescent acquisition at 55°C, followed by melt curve analysis with temperature increasing from 55 to 95°C with fluorescent readings acquired at 1°C increments. Three out of four best wheat control genes, encoding Actin, glyceraldehyde 3-phosphate dehydrogenase (GAPDH), cyclophilin and elongation factor 1-alpha (EF1a), were used for normalization of the expression as described by Vandesompele et al. (2002). Data from qRT-PCR experiments were expressed as copy number μg^–1^ RNA. All qRT-PCR assays were performed in three technical replicates.

## Results

### QTL Validation in Spica/Maringa Population

Spica (LMA-sus)/Maringa (LMA-res) was phenotyped over three successive seasons in glasshouse 1. The population was subsequently genotyped with several hundred publicly available SSR markers distributed across the genome. A locus in the sub-telomeric region of the long arm of chromosome 7B close to marker gwm344 explained 28, 42, and 43% of the observed variation in 2008, 2009, and 2010, respectively, with LOD scores of 11.9, 18.3, and 20.7 (Table 2).

**TABLE 2:** Results of QTL mapping for LMA in Spica (LMA-sus)/Maringa (LMA-res) DH in 2009.

LOD	% Phenotype explained	Marker	Additivity
13.67	35	wmc273	−0.12
17.79	42	gwm344	−0.13
12.26	35	wmc276	−0.12
10.46	31	barc182	−0.11

**FIGURE 1:**
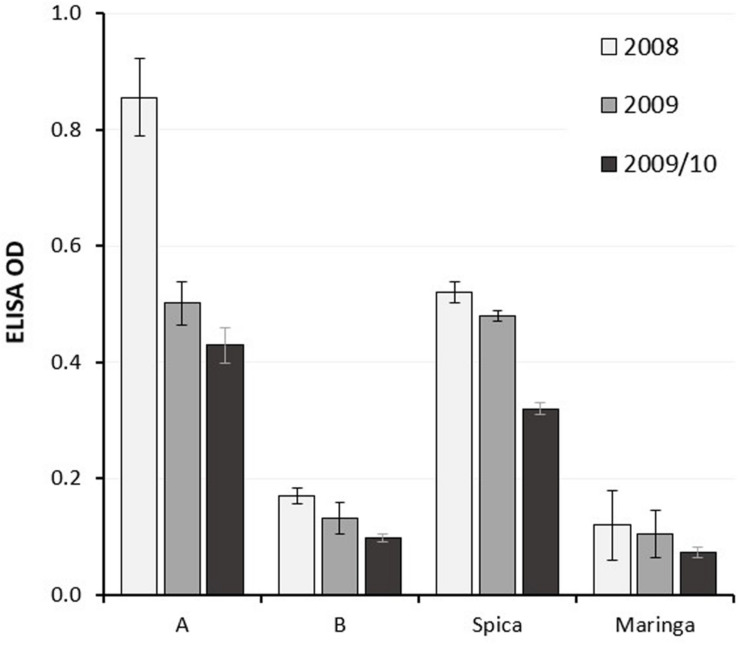
Mean LMA phenotype of sub-populations of lines from a Spica (LMA-sus)/Maringa (LMA-res) DH population grouped according to genotype at the LMA 7B locus compared with the parents. Data from three glasshouse experiments are represented as black, gray and light gray columns, respectively, with standard errors. A, Spica allele; B, Maringa allele.

Individuals in the population were grouped according to genotype at the 7BL locus using flanking markers and the mean LMA phenotype of the groups, each consisting of around 80 individuals, compared with each other and with the parents. As anticipated, groups having the Spica genome at the locus showed higher mean ELISA ODs (Figure 1), indicative of LMA-sus phenotypes.

### QTL Validation in Chinese Spring/Spica Population

The Chinese Spring (LMA-res)/Spica (LMA-sus) SSD population was phenotyped in glasshouse 1. Publicly available SSR markers on chromosome 7B (Schnurbusch et al., 2007) and KASP markers that were derived from SNPs in close proximity to Xpsr680 were tested for polymorphism between the parents and the population then genotyped to locate loci associated with variation in the LMA phenotype. SSR marker barc182 and KASP markers CAT, SPA, SOS, CPS2s8, and MYB co-segregated at a region that explained 29% of the observed variation and had a LOD score of 10.4 (Table 3).

**TABLE 3:** Results of QTL mapping in Chinese Spring (LMA-res)/Spica (LMA-sus) SSD.

Marker	LOD	% Phenotype explained	Additivity
barc32	5.62	17	−0.06
barc182, CAT, SPA, SOS, CPS2s8, MYB	10.24	29	−0.08
MATE1	9.39	27	−0.08
gwm146	8.31	25	−0.07

### Fine-Mapping of LMA 7B

For fine-mapping of the LMA QTL we used lines from crosses between Chinese Spring (LMA-res) and Spica (LMA-sus) as these provided a useful level of recombination across the critical region. In the Cranbrook (LMA-sus)/Halberd (LMA-res) population, we had encountered low levels of recombination within the target region. This was attributed to the presence of a tetraploid introgression containing *Bo1* (Pallotta et al., 2014) likely contributed by the durum line Cretan used to develop Halberd (Supplementary Figure 1)^6^. Similarly, a suspected introgression in Maringa that is positioned slightly distal to that found in Halberd may have contributed to the reduced recombination observed in the Spica (LMA-sus)/Maringa (LMA-res) derivatives (Pallotta and Cheong pers. Comm.).

Comparison of the LMA phenotype of 13 Cranbrook (LMA-sus)/Halberd (LMA-res) DH recombinant lines used by Schnurbusch et al. (2007) to fine-map the *Bo1* locus enabled additional marker development. Initially we made use of chromosome 7BL derived BAC-by-BAC sequenced contigs of the IWGSC wheat genome sequencing effort (Odd-Arne Olsen’s group, Norway) and the IWGSC scaffolds of RefSeq v0.4 once they became available. SNPs on chromosome 7B in close proximity to microsatellite marker barc182, were converted to KASP markers and used to screen 2160 Chinese Spring/Spica F_2_ seedlings for recombinants. Seedling leaves were genotyped to identify heterozygous recombinants, the seedlings transplanted to larger pots and the F_3_ seed harvested when ripe. F_3_ embryos were then genotyped to identify seeds that were either homozygous recombinant or homozygous non-recombined across the critical interval. Corresponding de-embryonated grains were pooled within their respective genotype group for ELISA analysis. Based on the results, the QTL region was reduced to a smaller interval (Supplementary Figure 2). Additional SNPs within this smaller interval were then used to screen a an additional 5000 Chinese Spring/Spica F_2_ seedlings to further reduce the size of the chromosome interval associated with an LMA phenotype. This was facilitated by manual ordering and assembly of several 7B scaffolds of CS IWGSC RefSeq v0.4.

**FIGURE 2:**
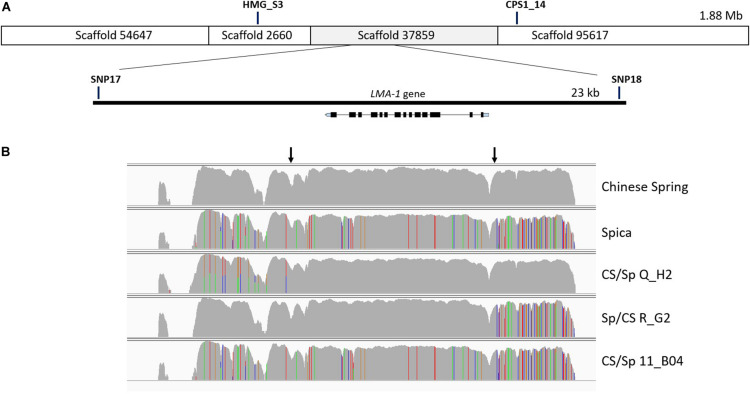
Fine mapping of the LMA region on chromosome 7B. **(A)** Manual assembly of CS IWGSC RefSeq v0.4 scaffolds into a 1.88 Mb contig for the LMA locus was followed by a second recombinant screen which allowed narrowing of the candidate region to 23 kb. Only one gene is located within the 23 kb region and its intron exon structure is shown to scale. The location of markers has been added for reference. **(B)** Graphical representation of read alignment in the 23 kb region visualized in Integrative Genomic Viewer (IGV). Reads were aligned to the CS IWGSC RefSeq v1.0 genome. SNPs are represented as colored vertical lines (red = T, green = A, blue = C, orange = G). The top two rows represent the parents of the mapping population, the bottom 3 rows show the genotypes of 3 recombinant lines, two of which show a recombination breakpoint, indicated by arrows. Q_H2 and R_G2 are phenotypical LMA-res, 11_B04 is LMA-sus. http://software.broadinstitute.org/software/igv/.

Based on a comparison of the LMA phenotypes of the recombinants, the interval was reduced to a segment of just under 23 kb (Figure 2A).

### Sequence Capture and Identification of a Candidate Gene Underlying the LMA-1 Locus

The absence of genome information for key LMA resistant and susceptible cultivars was the motivation to attempt sequence capture of the 23 kb candidate region as a means of determining allelic variation. Sequence capture was conducted for parental lines of the mapping populations, Chinese Spring (CS) (LMA-res), Spica (LMA-sus), and Maringa (LMA-res), and recombinants identified when screening the F_2_. Based on two recombinants, CS/Spica Q_H2 (LMA-res) phenotype and Spica/CS R_G2 (LMA-res) we were able to further reduce the LMA locus to a region of approximately 13.5 kb (Figure 2B). The recombination breakpoint in Sp/CS R_G2 delineates one border of the locus resulting in a change from Spica-genotype to that of Chinese Spring whereas the breakpoint in Spica/CS R_G2 delineates the other. In addition, the LMA phenotype of four recombinants derived from Spica/Maringa with break points adjacent to, but not within, the 23 kb interval also correlated between genotype and phenotype. Supplementary Figure 3 shows the allelic variation based on sequence capture for the Spica (LMA-sus)/Maringa (LMA-res) recombinants and their parents. Within the 13.5 kb interval, we identified by blastn and blastx searches against the NCBI nr database only one gene (from here on designated *LMA-1*). This gene showed sequence similarity to *ent*-copalyl diphosphate synthase-like proteins. The LMA-1 protein’s amino acid sequence contained catalytic motifs previously reported in plant CPS enzymes. These included an aspartate-rich DxDD that acts as the catalytic (Bronsted) acid (Prisic et al., 2007) as well as a histidine – asparagine dyad (conserved as LHS and PNV motifs) that help form the catalytic base (Lemke et al., 2019). A histidine present in many CPS enzymes associated with GA biosynthesis has been hypothesized to act as a biochemical regulatory switch (Mann et al., 2010). In the LMA-1 sequence, this histidine is replaced by arginine (Supplementary Figure 5). The presence of arginine appears to be typical of many CPS proteins associated with secondary metabolism. While deducing the intron-exon structure and protein sequencing from sequence alignment to related genes we discovered that Chinese Spring has a mutation that leads to premature termination of translation (Supplementary Figures 4A,B). In Chinese Spring, the stop codon occurred between the LHS and PNV motifs of the histidine-asparagine diad and before the DxDD motif (Supplementary Figure 5). As a consequence, the truncated protein produced in Chinese Spring would lack the enzyme’s catalytic site. In contrast, Spica contains a complete open reading frame (ORF), 828 amino acids in length, with an arginine at amino acid position 316 (codon: AGA where there is TGA in CS) (NCBI GenBank accession no. MW188549). Compared to a fully translated Chinese Spring sequence there are two additional non-synonymous amino acid changes [(CS) Ile 26 - > Val and (CS) Gly 620 - > Ser] (Supplementary Figure 5). Protein sequence comparison to previously published *ent*-copalyl diphosphate synthases (CPS) (Wu et al., 2012) showed that *LMA-1* represents a novel member of the CPS family in wheat (Supplementary Figure 6).

**FIGURE 3:**
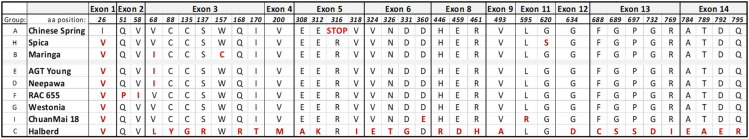
Polymorphic non-synonymous nucleotide changes leading to amino acid changes in the different allele groups. Differences in amino acids and their position in the deduced LMA-1 protein sequence are indicated. Outliers are highlighted in red.

The analysis of the capture results for Maringa identified a complete ORF with Arg (AGA) at amino acid position 316 and two non-synonymous substitutions of which Trp (157) -> Cys was notable due to the different chemical properties of the amino acids (Exon 3, Figure 3). A tryptophan in LMA-1 from Spica (LMA-sus) is replaced by a cysteine in the variety Maringa (LMA-res), a change that potentially might affect protein structure and function. The distance from the active site along with the buried nature of the side chain could be consistent with a structural role. Given the highly exothermic nature of the catalyzed reaction there is a requirement for structural rigidity of the CPS enzymes (Poralla, 2004). To further investigate this we located Trp (157) in the sequence alignment of monocot and dicot diterpene and triterpene cyclases generated by Köksal et al. (2011) (Supplementary Figure 4) and found it to be conserved across all 28 proteins of the alignment. This was also supported by sequence alignment of 101 Angiosperm sequences related to *LMA-1* in Phytozome V12^7^. We employed several variant effect prediction programs, including Provean and PredictSNP to examine the consequence of the W157C substitution and noticed that all programs suggested that the change could be deleterious (Supplementary Table 3, red box). Subsequently, the tertiary structure of Spica LMA-1 was *in silico* modeled with high confidence by Phyre^2^ onto the *ent*-copalyl diphosphate synthase of Arabidopsis (Köksal et al., 2011; Szymczyk et al., 2020). Overall, the sequence identity between LMA-1 and the Arabidopsis CPS was 54% and most of the protein was modeled except for the first 93 amino acids. The indication of high mutational sensitivity for W157 by Phyre^2^ encouraged us to submit the Spica model to Missense 3D for evaluation of Maringa’s Cys substitution at this position. No structural damage was predicted by Missense 3D. Nevertheless the Spica and Maringa models were compared. The W157C substitution potentially leads to a reduction of neighbor residues within 4Å (Supplementary Table 5) and also a change in polar interactions (Supplementary Figure 7A). Furthermore, a superimposition of the Maringa structure onto Spica’s showed changes of the side chains angles of neighboring amino acids and specifically a loss of interaction to Ala-120 (Supplementary Figure 7B).

### *LMA-1* Gene Expression During Grain Development

**FIGURE 4:**
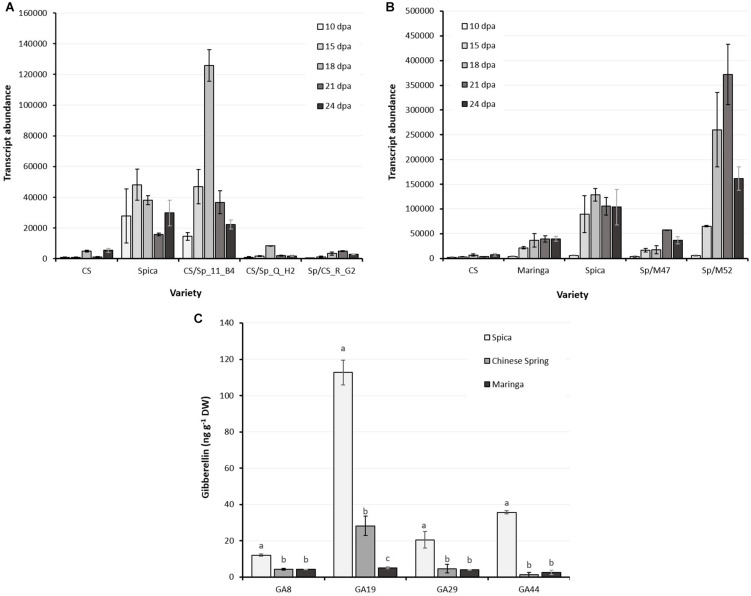
*LMA-1* transcript abundance in developing wheat grains sampled at 10, 15, 18, 21, and 24 days after anthesis. **(A)** Chinese Spring, Spica and recombinants from CS/Spica. Spica and CS/Sp_11_B04 are LMA-sus, all others LMA-res. **(B)** Comparison between Chinese Spring, Maringa, Spica and two progeny from Spica/Maringa with Spica alleles (Sp/M52) or Maringa alleles (Sp/M47). Error bars indicate SEM calculated from 3 biological replications at each sampling time point. **(C)** Gibberellin concentrations in developing grain (26 days post-anthesis) of Spica, Chinese Spring, and Maringa. Fourteen gibberellin species were quantified but only those above the detection threshold are presented. Shown are the means and SE of three replicate grain samples. Letters denote statistically significant differences between varieties (*P* < 0.05) for each gibberellin as determined by ANOVA.

*LMA-1* transcript abundance in endosperm sections of developing grains was determined by qRT-PCR at intervals from 10 to 24 DPA. Spica, and the recombinant LMA-sus line, CS/Sp 11_B04, had higher expression than CS and recombinant lines CS/Sp Q_H2 and Sp/CS R_G2 at all sampling times (Figure 4A). In addition, Maringa and the LMA-res line, Spica/Maringa47, selected from the Spica/Maringa DH population (Barrero et al., 2013) had lower transcript abundance during the early stages of grain development compared with Spica and the LMA-sus line Spica/Maringa52 (Figure 4B).

### Gibberellins

Gibberellins were quantified in developing grain of Spica, Maringa and Chinese Spring. Of the 14 gibberellins analyzed, only 4 gibberellins were detected in Spica; GA_8_, GA_19_, GA_29_, and GA_44_, and the levels of these gibberellins were all significantly lower in both Chinese Spring and Maringa (Figure 4C).

### Varietal Survey

The results reported above clearly demonstrated that some sequence variants of the *LMA-1* gene located on chromosome 7B are associated with resistance to LMA. The logical next step was to determine whether this model applied to other wheat varieties and/or whether there are other genetic loci associated with resistance to LMA.

Populations derived by crossing the LMA-res variety Halberd to nine separate LMA-sus varieties (BD159, RAC655, Seri 82, Reeves, Kennedy, ChuanMai18, Chara, Wyalkatchem and the synthetic hexaploid AUS33402), were phenotyped and genotyped using markers located close to the peak of the LMA 7B QTL region (Table 1). The percent variation in LMA phenotype associated with the 7B QTL varied from 17 to 52 while LOD scores ranged from 6.1 to 17.6. In each population, a Halberd 7B haplotype was associated with a low LMA phenotype and conversely, LMA-sus variety 7B haplotype was associated with a higher LMA phenotype.

In addition to the QTL on chromosome 7B, further QTL analysis of the Spica (LMA-sus)/Maringa (LMA-res) population identified two minor effect loci. One was located in the centromeric region of chromosome 3A between markers gwm5 and gwm2. This QTL explained 10, 6, and 10% of the variation in three separate experiments and gave LOD scores of 4, 2.1, and 4, respectively. Another locus located on chromosome 2D near markers gwm349 and wmc167 explained up to 5% of the variation.

**FIGURE 5:**
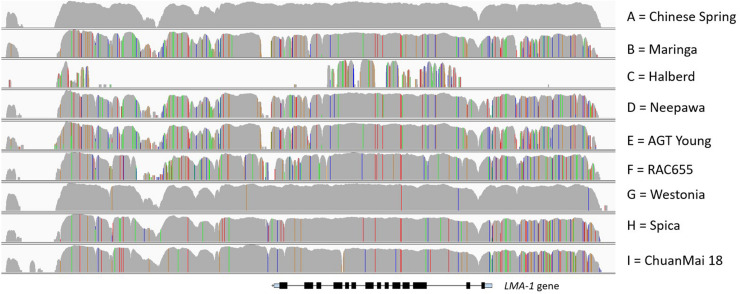
Visualization of SNP patterns of the different allele groups across the 23 kb candidate region. Reads were aligned to the CS IWGSC RefSeq v1.0. SNPs are represented as colored vertical lines (red = T, green = A, blue = C, orange = G). Row 2 Maringa, 3 Halberd, 4 Neepawa, 5 AGT Young, 6 RAC 655, 7 Westonia, 8 Spica, bottom ChuanMai 18. The approximate location of the *LMA-1* gene is shown.

For a comprehensive varietal survey, sequence capture was performed for an additional 48 varieties. Based on the SNP patterns derived from read alignments we were able to distinguish nine haplotype groups, labeled A to I (Figure 5), and identified unique SNPs for KASP marker development (Supplementary Table 4) which subsequently we used for large-scale genotyping.

Calingiri, and a Chinese landrace, SW95-50213, showed an identical genotype to CS; hence they carry the same mutation that results in a stop codon in exon 5 leading to a prematurely truncated protein. Group G members are genotypically closest to CS but contain the complete ORF with **A**GA (Arg) instead of the **T**GA mutation. In comparison, groups B, E and F are genotypically close, however, not identical, to each other, but show a large number of variants compared to CS.

The DNA sequence across the 23 kb region for *Bo1*-containing varieties (Group C, represented by Halberd in Figure 5) differed markedly from non-*Bo1* varieties to the point that read alignment was poor due to the large number of SNPs. We therefore assembled the *LMA-1* gene from sequence reads to deduce the protein sequence. Figure 3 gives an overview of the non-synonymous changes and their distribution across the 14 exons for each haplotype group.

Similarly, we examined positions with amino acid variations for the level of conservation across species and submitted the substitutions to variant prediction programs. We also checked the mutational sensitivity according to the predictions by Phyre^2^ after *in silico* modeling. However, as shown in Supplementary Table 4, for the majority of the changes there seems to be no significant impact on protein structure. At position 595, we found that Leu is conserved in around 80% of the 101 members of the Angiosperm gene family 93520577. Leu is substituted by Arg in ChuanMai 18 and this substitution is predicted to be ‘deleterious’ to the protein. However, this prediction was not consistent with the genetic information. In two experiments involving Halberd (LMA-res)/ChuanMai18 (LMA-sus), ChuanMai18 alleles at the LMA 7B locus explained 28 and 38% of the variation, respectively.

The Halberd sequence is quite distinct from that of Spica with many amino acid changes, several of which were predicted to affect the protein structure. These include V324E, the Valine at this position is >90% conserved among the 101 members of the *ent*-copalyl diphosphate synthase gene family, D331G (D, 100% conserved) and V493A (V, >70% conserved). All predicted deleterious substitutions were submitted to Missense3D for structural analysis. Changes in protein structure were observed in all cases (Supplementary Table 5 and Supplementary Figures 9–12).

### Varietal Survey – Genotyping and Phenotyping

A collection of 212 wheat varieties was genotyped for *LMA-1* using a set of SNP markers unique for each haplotype group and phenotyped for LMA (Supplementary Table 6). Included were most of the varieties that had been utilized for gene sequencing, the majority of modern Australian wheat varieties, and a selection of Canadian and European varieties. Due to space limitations, a smaller representative set of 153 lines was included in the summer screening in glasshouse 1. A subset of 76 varieties was also phenotyped in glasshouse 2 under conditions described in Derkx and Mares (2020) that promote LMA phenotype without the cool temperature shock.

**FIGURE 6:**
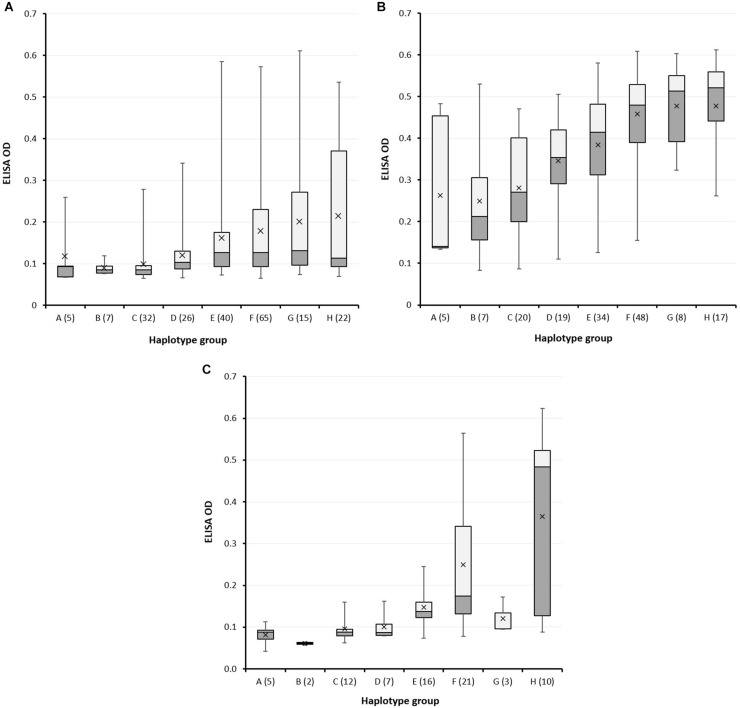
High pI α-amylase content in grain of wheat varieties carrying different *LMA-1* alleles. **(A)** A large collection of wheat varieties was grown in glasshouse 1 in winter for screening using cool-shock induction; **(B)** a subset was grown in glasshouse 1 in summer for screening using cool-shock induction; **(C)** a subset of 76 varieties was grown in glasshouse 2 using continuous cool daily maximum temperature but no cool shock. The box plot indicates the minimum, first quartile, second quartile (median), third quartile, and a maximum of each allele-group; the horizontal line within the rectangles is the median and the x symbol indicates the mean. OD = optical density. Haplotypes are represented by capital letters along the *x*-axis with the number of varieties in brackets. Haplotype I was excluded from the figure because only two varieties were available. Varieties, the allele they carry, and their ELISA ODs are listed in Supplementary Table 6.

In the first round of screening, the majority of varieties did not express LMA (the median OD was only 0.108), yet variation in LMA phenotype could be observed between haplotype groups. Wheat varieties with alleles A, B or C displayed lower LMA expression compared to the other alleles, as indicated by both lower median and mean α-amylase protein content (Figure 6A). The second round of screening during summer resulted in both higher levels and frequency of LMA expression (Figure 6B; median OD of 0.416), but varieties carrying the A, B and C alleles on average maintained the lowest grain α-amylase protein content. The Spearman rank correlation (r_*s*_) for the comparison of rounds 1 and 2 was 0.4390. The A, B and C alleles possessed by Chinese Spring, Maringa and Halberd, respectively, were consistently associated with reduced LMA expression in worldwide wheat germplasm. In the subset of 76 spring genotypes that were grown in glasshouse 2 and screened under constant cool temperature without a cool shock, while not strictly comparable with the cool shock experiments, results were similar to the winter screen in 2018 (Figure 6C) with a Spearman rank (r_*s*_) correlation of 0.7087. Derkx and Mares (2020) reported a strong relationship between expression under these glasshouse conditions and in the field. By comparison, the Spearman rank (r_*s*_) correlation for round 2 versus round 3 was 0.4712, similar to that for round 1 versus round 2. Overall, varieties that showed an LMA-res phenotype in one environment generally were at the lower end of the range in the other environments.

Notably, within haplotype groups D - H, irrespective of growing environment, there was a broad range in LMA phenotype which strongly suggests the involvement of alternative genome regions in some LMA-res varieties. For example, the 23 kb sequence for eleven varieties from SNP haplotype group F was shown to be identical yet, within this subset, LMA phenotype ranged from low (Janz, EGA Gregory, Sunvex) to high (RAC655, BD159, Seri 82, Maris Huntsman). Similarly, in haplotype group H, Hartog and AUS1408 (consistently LMA-res) have identical sequences across the region to Spica and Chara (LMA-sus).

### *LMA-1* Gene Expression Survey

*LMA-1* gene expression was examined by qRT-PCR. Primer pairs specific to either *Bo1*-containing or non-*Bo1* genotypes were used to account for their substantial sequence differences. Accidental amplification from DNA was excluded by having one primer spanning an intron, and careful analysis of primer design ensured homoeologue-specific amplification.

**FIGURE 7:**
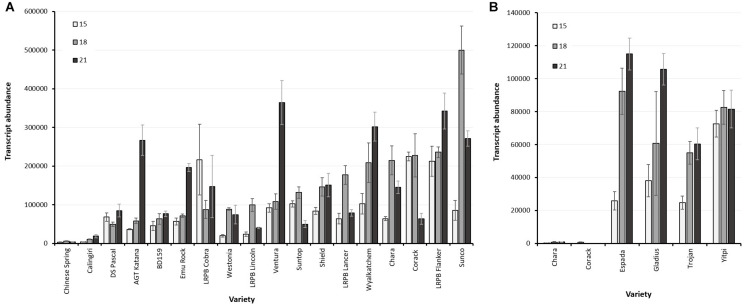
**(A)** *LMA-1* transcript abundance in developing wheat grains sampled at 15, 18, and 21 days after anthesis of non-*Bo1* varieties. Error bars indicate SEM calculated from 3 biological replications at each sampling time point. **(B)** *LMA-1* transcript abundance in developing wheat grains sampled at 15, 18, and 21 days after anthesis in *Bo1*-varieties using specific primers. Error bars indicate SEM calculated from 3 biological replications at each sampling time point. Chara and Corack are non-*Bo1* and were run here as negative controls for amplification.

*LMA-1* transcript abundance within the non-*Bo1* group varied from very low in varieties, such as Chinese Spring and Calingiri, to high in LMA-sus varieties such as RAC655, Chara and Kennedy (Figure 7A and Supplementary Figure 8). In these latter varieties, transcript levels increased from 10 days post-anthesis, reached a peak by 15 or 18 DPA with no further increase after 21 or 24 DPA. Across a broader range of varieties examined, however, there did not appear to be a clear relationship between *LMA-1* transcript abundance and LMA phenotype (Table 4). Abundant transcripts were also observed in *Bo1*-containing cultivars such as Espada, Gladius and Trojan (Figure 7B) when primer pairs specifically designed for Bo1-containing varieties were employed. Some varieties with high *LMA-1* transcript levels (e.g., Hartog, Flanker, Sunco, Suntop, and Ventura) and Janz (data from a separate experiment and not shown) did not show an LMA phenotype under the conditions used to grow the plants. Of particular interest was a comparison between RAC655 (LMA-sus) and the LMA-res variety Hartog where resistance does not map to 7B. Both varieties had similar high transcript abundance profiles despite having phenotypes at opposite ends of the phenotype (Supplementary Figure 8). Furthermore, transcript abundance did not appear to be enhanced by 3 days of cool temperature shock. Simple correlation coefficients (r) between transcript abundance at three time-points (15, 18, and 21 DPA) and LMA phenotype (ELISA OD at harvest-ripeness) ranged from 0.121 to 0.16.

**TABLE 4:** LMA phenotype of genotypes used in the variety survey of transcript abundance during grain development.

Genotype	ELISA OD	SE	Genotype	ELISA OD	SE
Chinese Spring	0.075	0.001	Shield	0.115	0.001
LRPB Trojan	0.088	0.001	LRPB Lancer	0.317	0.000
Calingiri	0.090	0.010	Wyalkatchem	0.417	0.040
DS Pascal	0.123	0.018	Chara	0.440	0.014
AGT Katana	0.227	0.093	Corack	0.101	0.016
BD159	0.582	0.021	LRPB Flanker	0.100	0.007
Kennedy	0.231	0.046	Sunco	0.100	0.007
Emu Rock	0.121	0.010	Gladius	0.055	0.001
LRPB Cobra	0.282	0.019	Yitpi	0.155	0.037
Westonia	0.167	0.042	Espada	0.061	0.004
LRPB Lincoln	0.082	0.005	Hartog	0.095	0.012
Ventura	0.160	0.027	RAC655	0.425	0.032
Suntop	0.151	0.010			

*LMA was determined as α-amylase protein by high pI-specific α-amylase ELISA on harvest-ripe grain samples.*

## Discussion

Genetic evidence from populations derived from crosses involving LMA-sus varieties and LMA-res genotypes Halberd, Maringa or Chinese Spring implicate a locus distal on chromosome arm 7BL in LMA phenotype. In retrospect, it was fortuitous that these particular resistant parents were used. In each of these varieties, the *LMA-1* protein sequence was altered (Halberd and Maringa) or prematurely truncated (Chinese Spring).

Varieties with the same *LMA-1* allele as Chinese Spring, Maringa or Halberd, were less common than varieties with a functional *LMA-1* gene. This suggests that a functional *LMA-1* gene may be the more common wild-type condition even though it is not always associated with LMA susceptibility. This represents a genuine paradigm shift since until now LMA was considered a genetic defect present in only a few wheat varieties (Mares and Mrva, 2008, 2014). The mutations and the introgression appear to be very useful sources of resistance to LMA and the diagnostic markers developed in this research will be useful to wheat breeders aiming to reduce the risk of LMA occurring.

Phenotypic screening of a variety panel confirmed that certain mutations in *LMA-1* are associated with reduced LMA susceptibility. This is particularly noticeable in haplotype groups A, B and C. Yet the wide range of LMA phenotypes within other haplotype groups is strongly suggestive of LMA being a complex trait, in other words, the *LMA-1* genotype on its own only partially explains the LMA phenotype of a variety and there are likely to be some other genetic loci involved. Furthermore, the interaction of genotype with the environmental triggers of LMA (temperature) is complex, resulting in a large range of phenotypes even within plants of the same variety (Derkx and Mares, 2020). In this context, several QTL for LMA or low falling number have been described in wheat (Mrva and Mares, 2001b; Mrva et al., 2009; Emebiri et al., 2010; Tan et al., 2010; Mohler et al., 2014; Zhang et al., 2014; Börner et al., 2018; Martinez et al., 2018). It is very likely that some of these QTL interact with *LMA-1*, potentially explaining the wide range of phenotypes within the different *LMA-1* haplotype groups. For instance, despite appearing to be alleles encoding a functional protein, groups D and E displayed an average LMA phenotype intermediate to that of Spica and Chinese Spring. These two groups contain a high proportion of Canadian varieties, which on average had lower LMA phenotype than Australian wheat cultivars. There are also a number of Australian varieties, for example, Hartog and EGA Gregory, with known high resistance to LMA but with an identical *LMA-1* sequence to very susceptible varieties within their respective haplotype groups. These LMA-res varieties presumably have their basis in genetics, although a role for epigenetics cannot be excluded. The *LMA-1* gene was not sequenced for all of the 216 wheat varieties and potentially some of these varieties could contain additional SNPs that are not present in the varieties that were sequenced.

Based on its sequence, *LMA-1* was annotated as a putative *ent*-copalyl diphosphate synthase (CPS). Two biological roles have been described for CPSs thus far: catalyzing the first dedicated step of gibberellin biosynthesis and initiating the production of labdane-related diterpene phytoalexins that are involved in plant defense (Zi et al., 2014; Murphy and Zerbe, 2020). *TaCPS3*, which is located in close proximity to *LMA-1* on chromosome 7B (marker CPS1_s14 in Supplementary Figure 2) and is in sequence most similar to *LMA-1*, has been described as the CPS involved in gibberellin biosynthesis in wheat (Toyomasu et al., 2009; Wu et al., 2012). The amino acid sequence for *LMA-1* from the LMA-sus variety Spica contained the aspartate-rich DxDD and the histidine-asparagine (H-N) dyad catalytic motifs that are known features of CPS enzymes involved in GA biosynthesis (Prisic et al., 2007; Lemke et al., 2019). However, a histidine that has been hypothesized to act as a biochemical regulatory switch is replaced by arginine in LMA-1 (Arg_335_ in Spica). This histidine is conserved in CPS that are associated with GA biosynthesis whereas arginine is often found in CPS associated with specialized/secondary metabolism (Mann et al., 2010). Mann et al. (2010) used recombinant proteins to study the effect of the His/Arg substitution and found that, while both forms retained significant activity, the Arg-substitution was no longer inhibited by Mg^2+^ thus allowing for rapid production of secondary metabolites such as those involved in plant defense.

The reduction in the production of gibberellins in developing grains of Chinese Spring and Maringa compared to Spica suggests that the protein encoded by *LMA-1* is also capable of catalyzing the initial step of gibberellin biosynthesis. This does not prove that this is its main function, however, or that the changes in gibberellins are directly responsible for the differences in LMA phenotype. Since the analysis of both *LMA-1* gene expression and gibberellins was performed in de-embryonated grains consisting of starchy endosperm, aleurone and seed coat, it is not certain that gene expression, gibberellin biosynthesis and α-amylase production happened in the same tissue. Furthermore, Toyomasu et al. (2015) demonstrated that in rice the reactions catalyzed by different CPSs are in all likelihood mainly determined by their tissue localization rather than inherent differences between the enzymes.

Yet the role of the *Rht-B1b* and *Rht-D1b* dwarfing genes that encode the gibberellin-signaling DELLA protein (Peng et al., 1999) in reducing LMA expression has been well established (Mrva and Mares, 1996; Farrell et al., 2013; Derkx and Mares, 2020), giving more weight to the idea that gibberellins are involved in LMA. The role of gibberellins in activating high pI α-amylase expression during seed germination via DELLA and the transcription factor GAMyb (Gubler et al., 1995) also makes a similar mechanism during LMA an attractive hypothesis. Barrero et al. (2013) had already demonstrated that gibberellins were produced in LMA-sus doubled haploid lines from Spica/Maringa, but until now this could not be linked to the presence of specific genetic loci inherited from Spica. The four gibberellin species found in the developing grain of Spica (Figure 5) are all part of the 13-hydroxylated branch of gibberellin biosynthesis (Hedden and Thomas, 2012), suggesting that this is the dominant pathway in developing wheat grain of this variety. The presence of small amounts of the GA_1_-catabolite GA_8_ suggests that some bioactive GA_1_ must have been produced, but none was measured. Developing wheat grains have been shown to possess a unique GA1-oxidase enzyme leading to the biosynthesis of GA_54_ and GA_55_ as the bioactive GAs rather than the more common GA_1_ and GA_4_ (Gaskin et al., 1980; Lenton and Gale, 1987; Pearce et al., 2015). Therefore the presence of GA_55_ rather than GA_1_ would be expected. Unfortunately, no authentic standards for GA_55_ are commercially available, precluding its measurement in the experiments described here. Further research involving the application of gibberellin and gibberellin biosynthesis inhibitors to developing wheat grains with different alleles of *LMA-1* and *Rht-1* is required to resolve the role gibberellins might play in LMA expression and is currently underway.

The mechanisms and intermediates involved in the pathway between *LMA-1* and the synthesis of high pI α-amylase remain unclear. The data presented in this report could be interpreted as indicating that there are potentially several levels of control. *LMA-1* transcription during grain development in several varieties including Chinese Spring and Maringa was very low. Based on the comparison of Chinese Spring, Maringa and Spica, there appeared to be an association between *LMA-1* transcript abundance and LMA phenotype. However, when a more extensive range of genotypes was investigated the relationship could not be corroborated. While there appeared to be regulation of *LMA-1* transcription, a low abundance of transcripts did not necessarily result in a low or non-LMA phenotype. Transcription was detected from 15 days after anthesis, several days before the synthesis of α-amylase protein reported by Derkx and Mares (2020), and did not appear to be enhanced by a cool-shock that triggered or accentuated LMA phenotype in many genotypes.

No information is available on the next step in the pathway, translation of the *LMA-1* transcripts to produce *ent*-copalyl diphosphate synthase protein. In the case of the Chinese Spring, Maringa and possibly Halberd alleles, the protein would either not be produced (Chinese Spring) or its function likely impaired (Maringa and Halberd). In earlier research, tall genotypes were not subjected to a cool-shock since it did not appear to be required for development of a phenotype. The cool temperature shock-induced LMA phenotype observed in Chinese Spring, Maringa and Halberd in the summer experiment in this study was therefore unexpected. The substantial difference in phenotype between the winter and summer experiments is consistent with previous experience gained from screening thousands of advanced breeding lines for wheat breeding companies over several years (Mrva and Mares pers. comm.). It is possible that the magnitude of the cool shock determines the phenotype. Further research is required to test this hypothesis. Derkx and Mares (2020) reported that results from summer experiments were not well correlated with field data. At least in the case of Chinese Spring, the stop codon in the sequence would result in non-functional protein irrespective of the cool shock. Possibly other genetic loci participate in the response to the cool shock and consequently an assay for *LMA-1* protein could provide critical information. It is not clear how much protein is required, how stable the protein might be, whether it accumulates, or whether there is a threshold that is required for a phenotype. Certainly, the possible causal link between *LMA-1* and LMA requires further investigation. This could be achieved using a number of alternative approaches including transgenics, TILLING, gene silencing and gene editing.

The presence of genetic loci other than *LMA-1* has been reported previously (Mrva and Mares, 2001b; Mrva et al., 2009; Emebiri et al., 2010; Tan et al., 2010). The 3B QTL (Mrva and Mares, 2001b) and the 3A QTL identified during their study are both centromeric loci, possibly homoeologous, and both appeared to have a cumulative effect in combination with 7B. How they function and whether they are activated by cool temperature is unknown. Genetic loci that appear to be present in varieties such as Hartog and Janz are of considerable interest since these varieties accumulate presumed functional *LMA-1* transcripts but the subsequent pathway to induction of α-amylase synthesis is inhibited and a cool-shock does not normally result in an LMA-sus phenotype. Genetic control of resistance in these varieties is the subject of further investigation.

In conclusion, LMA-sus appears to be the wild-type phenotype although it is not clear what role it plays in grain development or why LMA-res does not appear to preclude the development of successful commercial wheat varieties. Genetic evidence implicated a gene located on chromosome 7BL in the observed variation in LMA phenotype and this gene (*LMA-1*) was subsequently identified as an *ent*-copalyl diphosphate synthase. Deviation from the wild-type, whether due to a reduction in *LMA-1* transcripts resulting in lower protein or synthesis of non-functional protein, results in useful resistance. Diagnostic markers for this resistance have been developed and could be deployed in breeding programs. In addition, there are alternate sources of resistance both within Australian and Canadian germplasm that could be characterized and exploited.

## Data Availability Statement

The datasets presented in this study can be found in online repositories. The names of the repository/repositories and accession number(s) can be found in the article/Supplementary Material.

## Author Contributions

The project was conceived by DM and the manuscript was written by DM, UB, and AD with input from the remaining authors. Populations were prepared and phenotyped by KM and DM who also conducted experiments to provide material for gene expression and gene sequencing. The variety survey and gibberellin experiments were carried out by AD. QTL discovery, fine-mapping, assignment of varieties to the different haplotype groups and gene expression studies were conducted by JC with input from MP. UB designed the sequence capture experiments, analyzed data, and supervised the bioinformatics work. NS analyzed NGS sequence data and assisted with the bioinformatics. All authors contributed to the article and approved the submitted version.

## Conflict of Interest

The authors declare that the research was conducted in the absence of any commercial or financial relationships that could be construed as a potential conflict of interest.

## Footnotes

1 https://nrc.canada.ca/en/research-development/products-services/technical-advisory-services/plant-hormone-profiling

2 www.lgcgroup.com/our-science/genomics-solutions/genotyping/kasp-genotyping-chemistry

3 https://www.bioinformatics.babraham.ac.uk/projects/fastqc/

4 http://www.sbg.bio.ic.ac.uk/∼phyre2/html/page.cgi?id=index

5 https://pymol.org/2/#page-top

6 http://wheatpedigree.net/

7 https://phytozome.jgi.doe.gov/pz/portal.html#!showCluster?search=1&detail=0&method=5197&searchText=clusterid:93520577
