# Supplementary figures and images for: A Major Locus on Wheat Chromosome 7B Associated With Late-Maturity α-Amylase Encodes a Putative ent-Copalyl Diphosphate Synthase
Source: Front Plant Sci. 2021 Feb 26;12:637685. doi: 10.3389/fpls.2021.637685 (PMC7952997; doi:10.3389/fpls.2021.637685)

## Slide 1
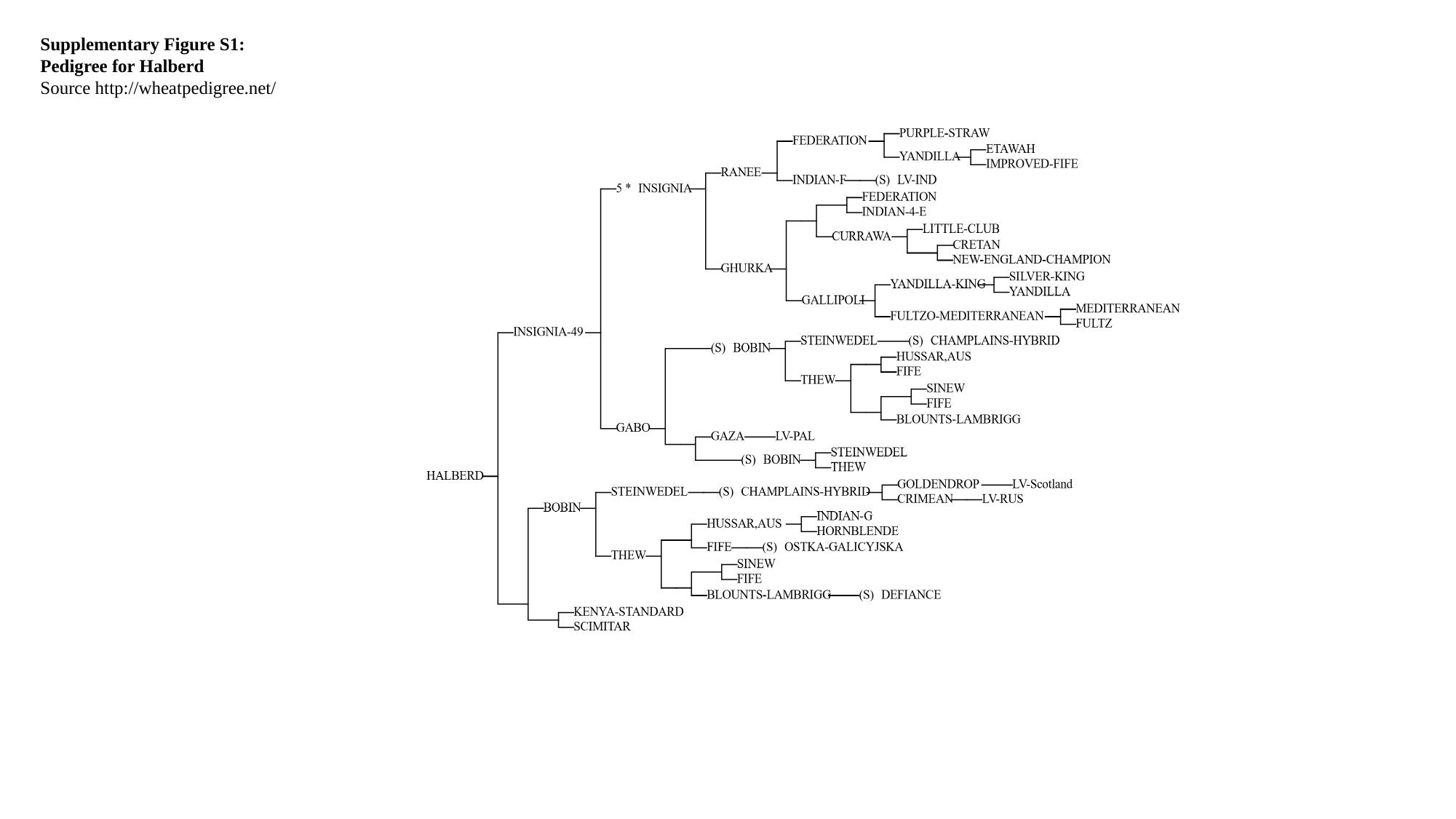

Supplementary Figure S1:
Pedigree for Halberd
Source http://wheatpedigree.net/

Supplement: Supplementary file 2 [file Presentation_1.pptx]

## Slide 1
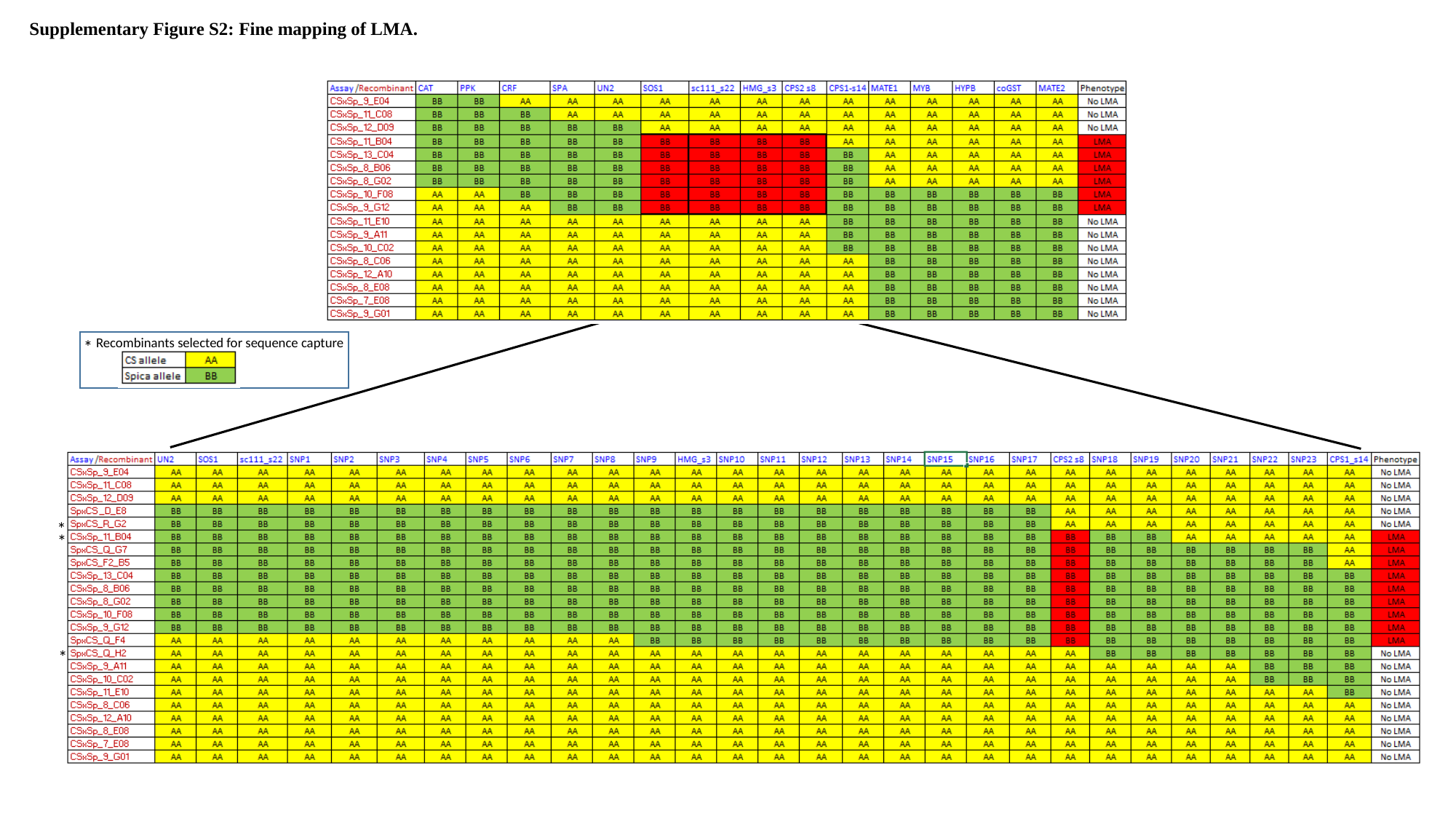

Supplementary Figure S2: Fine mapping of LMA.
Recombinants selected for sequence capture
*
*
*
*

Supplement: Supplementary file 3 [file Presentation_2.pptx]
