# Supplementary material for: A Major Locus on Wheat Chromosome 7B Associated With Late-Maturity α-Amylase Encodes a Putative ent-Copalyl Diphosphate Synthase
Source: Front Plant Sci. 2021 Feb 26;12:637685. doi: 10.3389/fpls.2021.637685 (PMC7952997; doi:10.3389/fpls.2021.637685)
Supplement: Supplementary file 4 [file Presentation_3.pptx]

## Slide 1
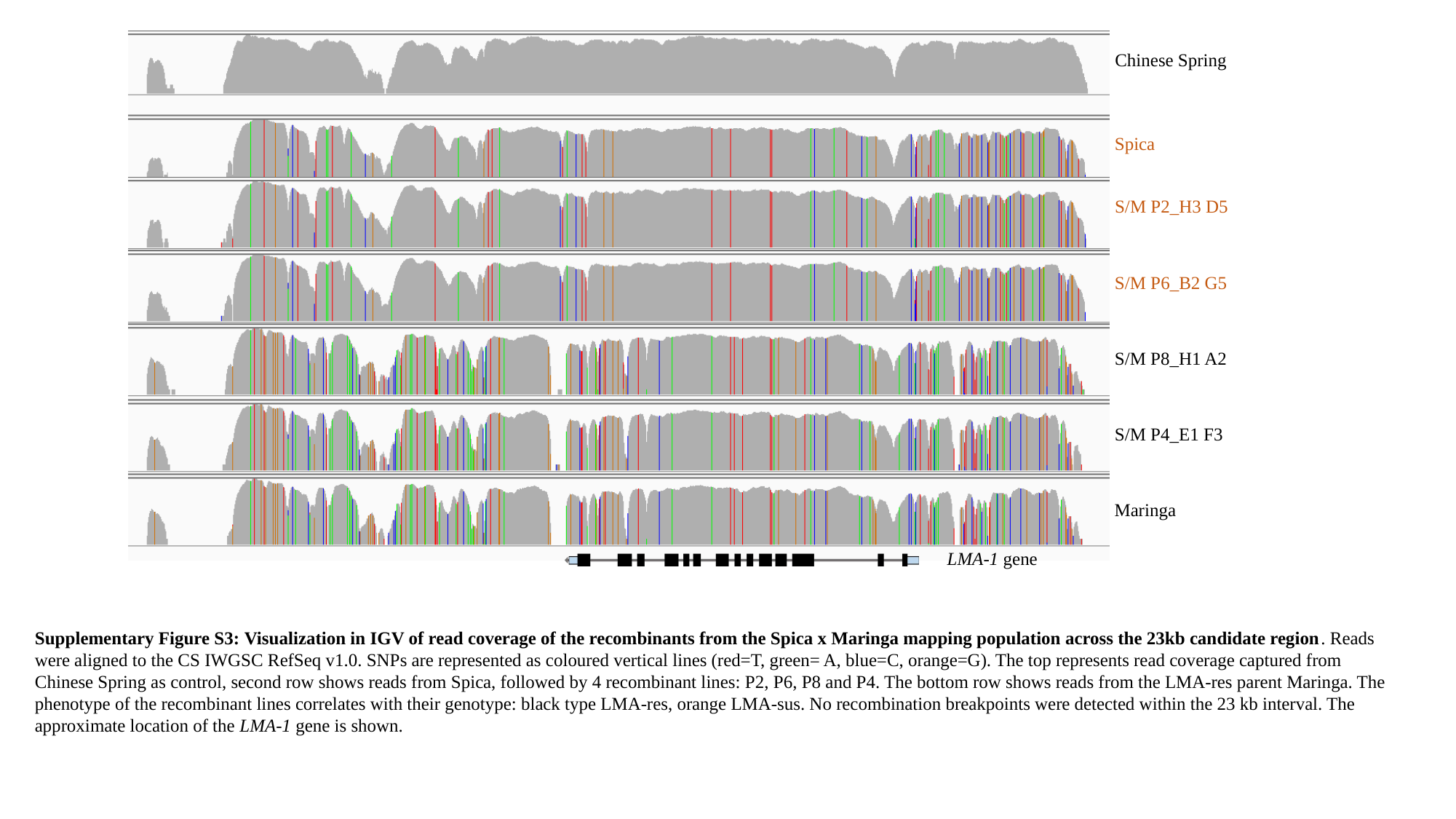

Chinese Spring
Spica
S/M P2_H3 D5
S/M P6_B2 G5
S/M P8_H1 A2
S/M P4_E1 F3
Maringa
LMA-1 gene
Supplementary Figure S3: Visualization in IGV of read coverage of the recombinants from the Spica x Maringa mapping population across the 23kb candidate region. Reads were aligned to the CS IWGSC RefSeq v1.0. SNPs are represented as coloured vertical lines (red=T, green= A, blue=C, orange=G). The top represents read coverage captured from Chinese Spring as control, second row shows reads from Spica, followed by 4 recombinant lines: P2, P6, P8 and P4. The bottom row shows reads from the LMA-res parent Maringa. The phenotype of the recombinant lines correlates with their genotype: black type LMA-res, orange LMA-sus. No recombination breakpoints were detected within the 23 kb interval. The approximate location of the LMA-1 gene is shown.
