# Supplementary material for: A Major Locus on Wheat Chromosome 7B Associated With Late-Maturity α-Amylase Encodes a Putative ent-Copalyl Diphosphate Synthase
Source: Front Plant Sci. 2021 Feb 26;12:637685. doi: 10.3389/fpls.2021.637685 (PMC7952997; doi:10.3389/fpls.2021.637685)
Supplement: Supplementary file 5 [file Presentation_4.pdf]

GGATTTCCCTACGCGCGCGCGCAGATCATCCACGCGCGCGCATTCCTTTTTCCATTCCGCTC
CGGAACAGCAGAATCTAGAGAAAGTGAAGCGGATCAGTTGCCGGCTGAAGAAAAAGGATCTG
GCTCCCTTCCCGGACCGGCCATGGCGTGGCATCTTGGCGTGACGGGGGCGGAGCATCTCGCC
CTCGCCCTCACACCACCCGTTCACAAAGCGAACGTTTCTAAGCTTTTTGTTTTGAACACTGC
CAGAGGCCTTTGCCGTGCAGGAATCATTCCCTTTATCCCACACTCGTCTCTCGCTTTTGTTC
TTGTATCTATGTATGTATCCTTGTATATATGCATTGCAATTGCACTGCACGGCACACATACA
TGTCCCTCCCGGATAACCCCGCGCCGTTCTCGTTTGTTTCGCTCATTCCTTAGCTGCCACCC
TTGCCCCTTCTTTGCTTTATCATCATCATCATCATCATCATCATCCTTAACACTCGGCGTTG
*GGAGGGGCTGGAGGAGTGGTGTTCGTCGCGGATTGAGGAACGTGCATTGCCGCAGCTAGC*AT
GCAGCTACACCTCTCGCCGCCGGCGTGGGTGCCATCGGGCTACGGCCACGGACCGCGGCCGC
GTGCTCTCGTCATCAAAGGTGTTCGGTCGATGTCGCTTTCTGTCGTGCTTTTCGCGCGAGGC
GCTGGTTTTCCCTGTCGTACGATGTACCGTGTGCACCGTGATTAATTATGGGGGTGCACCAT
GTGTGTCTTGCGATTCTTCGTGTGCCGGCCACGCTCTGCGGGGATACGGCTGCAGGAGGTCT
TGTCTGTCATTCGCACACGCGAATTGTGATGCCTGCAGCCCGCGACCGTTTCGCCCTGCCCA
CCGCAGACAAGTGTCGGCATGCTGTGTTGAGTTTGAAGCGAGACCGACATGGGCACTTGTCG
TGAGGTTGATTCTGATCACTTTCTCTTTGAACAATCAGGTCCATGCCGTTTCCGCGGCAAAG
GAGATGCTGCCCTTGTGGAAACCGCCGGCTCGCGTGTCGCTCTCCAGATTGCGCAGGCCACC
AGCGTATCCAGTGCTAATGGTATCGTTGCCTTGACTTGGTCTGGGACCCGGTGATCTTGTGT
TTGTTTTATTCTTATGTTCTCTCGATCGCTCGCTTTAGTGTCACTATCCACTGTTAGCTAGC
ACTGGGCAAGTATCATATACATTACTGATTAACTAATTAATCTCAGTCGGCCATCTCCCGTT
TCTACTGTCTGTTATTTTGCTATCTGCTATTATGCCAGCCTATGTACTCCAAGCTGTAGGCA
GCTAATTAGTACATATATTGTTTTAGTCCTTTTTATTATGTTCCTCTTTAGCGACTACACGT
CACATAATTAACCTATTTGGAGCTCAATTACACATGCTGCTACTCGTTTACCCGAGGAACGA
ATGGCCTCTGGTGAGTTGGCACGTGCTATACCAATTATAGCACACGTAATTAGCAGGTGAAA
CATGAACAAATGATCAGTTAACGAATCAGGAGTGGATAGGACCCGGGTTTTCGGAACACACA
GCACATTAATTAGCAGGTGAGACAAGAATAAATTATCCCTCAACGTCTCTAGGTCCTTTTTA
CAAAACGACACGACATGCCATTTGTCGCCAAGGGGAATCGCAACGATACTGATAGAGTGCAC
AGAAACACGAGAAAAGGGTATACTAGAGAGAAGGGTAGGATTAAGATTTTTTCTTTTGGAGC
ACACAAAACGTTAACTAGTAGGCGAGACAAGAATAAACGGTCCGTCAACGACTATTTTTTTG
GCACCACGAAGAGAAGGAACCCGAACCTATATGAAGTTCACTTTTGAACAATGATGTAGCTT
TCTTATCAGATCCCAAAGCATCAACTATACAAAAGAATGCTTCTGCAGTTTAAAGTCAAGTC
TGATGATAGAATGATTCTCGACATACTATTGTTCTCGTTATCATCACTATAATACTGTCACC
GCCATCGTTCTTTCATCAGGTAATAAAATCCTAGGTGAACTTAACCCAATCTCAGTCTGTAT
AACTTGAATACAATTTTACAATCACGTAAACTGACCTGGAGCTGAAATTTGGTAACATGTTA
ATGCACATCGAGGCATTATTATTCTAGTTAATTCAAAATGGATGCGATGTCTTGCGCTGGAT
ACATTTCATCATTTGTGTGTTTCGATGTACCTGCAGGTTTGCAAACGAACGTTGTCAGAAAC
GATATTCAGGTTCTTGAACGGACTGAGGAACAATGTGAACTCGACGACTGTTGTGTGATTCC
CGGGACAGAGTTTGAGCAGCCACTGGTCGACCAAGTGAGGATGATGCTGGGGTCGATGAGTG
ACGGTGAGATCAACGTCTCGGCATACGACACCGCCTGGGTCGCTCTGGTGCCGAGTCTTGAT
GACTGTGACAGCCCCCAGTTTCCCACCACCCTCCGGTGGATTCTTGACAACCAGCTCCCAGA
CGGCTCCTGGGGAGACGCTGCCCTGTTCTCCGCCTATGACCAGGTCATTAACACCCTTGCCT
GTGTCGTGGCTCTTACAAAGTGGTCTCTTGGCCCTGATAAATGCAGGAGAGGTATTGCAAAG
CAACAAACTGACACACATAACTGTTCTGCTAAAGTACCAAATGAAGCAAGGTCTTTCAACTA
AATGTATGCATGATGCATCCTGGTTTTCTTTTCAGGGCTCTCTTTTCTGGAGGAGAACGTGT
GGAGGCTAGCTGAGGAAGACCTGGAGTCGATGCCCATCGGCTTCGAGATTGTGTTCCCTTCT
CTCCTGGAGGTGGCCAAGAGCTTGGGCATTGGGTTCCCGTATGACCACCATGCTCTGCAGCG
CATATATGCTAACAGAGAAGTGAAGCTCAAGAGGTACCCCATATATCCCCTTTCCTGCCAGA
GATGCAGATTACTTCACAGTTTTCCTAGTTAATCATTAATTACTTCGGTAGGATTCCGATGG
AGATGATGCACAGGATTCCAACGTCGATCCTGCATTCCCTTGAAGGATGCCCGGGGTGGAC
TGGCAGAAAATCCTTAGGCTCCAGTCTAGTGATGGGTCCTTCCTCTATTCTCCTTCGGCTAC
AGCCTGTGCTCTCATGCAAACCGGTGATGAGAAATGCTTCGAATACATCGAC**T**GAATCGTCA

AGAAATTCAACGGAGGTAAGAGGTCCGTGACCATAAACCACACTGATATTGGATGGCATTGA
GTTCTTTTTTGGGGTAGTTCCTGATCCCGTGTGCATTGTTCATCTTGATTGGCGCATTTCAG
TTCCCAATGTTTACCCGGTCGATCTCTTTGAGCGCATCTGGGCCGTCGATCGGTTGGAGCGT
CTTGGAATCTCGCGCTATTTCAAGCAAGAAATCAAACAGTGCTTGGACTATGTTCACAGGTT
TAATTTCTCCTTCATAACTGTGTGTTGTTAACAATAGCATGGTAATCTTAAACTTTTGGTCG
CTTCATGAGGAAGGTTTCTTCTAACCTCTTTACTCACGCTTTAGGCACTGGACTGATGAGGG
GATTTCCTGGGCGAGGAACTCCACTGTAATAGACGTGGATGACACATCCATGGCGTTCCGGC
TGCTGCGGCTACATGGATACGATGTCTCCCCTAGTATGACCCCGCTTTTTCATGCAAATCCA
AATATTGTATCGTGCTGTACATACTCCGCAGAGTAATAAATCATATCACAAACAAAACTAAC
ATTTTGATTAATTCACAGCTGTATTTGAGAAGTTTGAGAAGGACGGGGAGTTCTTCTGTTTC
GTGGGGCAATCAACACAAGCAGTCACTGGGATGTACAACCTGAACAGGGCCTCTCAGGTAAG
GTTCCCCGGAGAGGACTTGTTGCAGCATGCAGGGAGATTCTCGTATGAGTTCCTGAGAGAAA
GGGAAGCCCGTGGCACGATTCGAGACAAATGGATCATTGCTAAGGATCTACCAGGCGAGGTA
AACACGTCCGTCCTGCCAATCAGTCGGTTTAGATGGACTAGGAAGTAAACATAACTCTCACA
ATCTTTGGCTTGATTGCTGATGACACTTGAGAGTTTTTTTTTGAAACCATTAAAGAATTATA
CTTGATATTTCCATATACAATAGGACTATTTAAACACTCTCTTGTTAACATATTAAAAAAAC
TTATTATCTTATTTTTGTGAAACAAACTAACCTTATCTGGATACGAACAAGAATATAAGTAC
CTTTATGTATGATTATAGGTAAAGTATACACTGGACTTCCCATGGTATGCAAGCTTACCGCG
TGTAGAAGCAAGAGTCTACCTCGATCAATATGGTGGTGATAATGATGTCTGGATTGGAAAGA
CACTCTACAGGTAAAAAATGGTTGAATTCCAACAATTGGTTCATTCCATACTTGTTGAGGAA
AGATCCCAGAGTTGATATGCTGAATGGTTTTGGCAGGATGCCACTTGTGAACAACAACACCT
ATCTTGAGTTGGCAAAGCGTGATTTCAATCGCTGCCAAGTCCAACATCAGCTTGAGTGGCAT
GGCCTACAAAAGTACTCATTAATCTCATGTAACATCTTGTTAATTACGTCCTATTTTTCCTG
CATAACATGTCTTCCTATGTATTTAGGTGGTTTACTGAGAATGGCCTCGAGACTTTTGGGGT
GACTTTAAGAGATGTTTTGAGAGTTTATTTTCTAGCCGCCGCTTGCATTTTCGAGCCAAGCC
GTGCCACCGAGCGACTTGCATGGGCCAAGGTGTCAGTGCTGGCCAACATTATTACTAAATAC
CTTCATAGCGATTTGTCGGGTAATGAAATGATGGAACGGTTTATGCAAGGCGGTATCTATGA
AGGAAATAGTAATATATCATGGTGAGCATATTAACTGAGCATCGCTACTCAATCTAAACCAT
CACACCAAATGATAAATATTTACATGCTTAACTGGATCCCTTTGGCCAATTGTTCTTTTGAT
AAGAAAAATAGCAAATTAATATACTAGTAATTTGGTAGATGATTCCCACAGGTGGACAGTTG
TGATGCAAACATAAAATCCACTATCTTAGAAATTAAGACCTTCAAACTTGTATATGTAGCTT
TTTTTTTGCTTACGAGGGATGATGTAATCTTTAAACACTGCTATTTAATCCATTGCTGTGCC
ATTATGAAAACATGGACTTCCGATTATAATGTCTTTTCATTTTTTTAATATGGTTCACATGA
CACAATTTGTCCATCCAGGCATAAAGGAGGTGCAAAAGAGGACATTCTTGTCGGGGCATTTG
AGCAACTTATTGATTTATTGGCACAAGAGGCACTACCTGTTGGTGAAGGACCAGTGTACATC
AACAATTTGTTACGCTGTGCTGTAAGTTGAACGACCAATTGCCCCTTTTCTTAGAGTTATTA
ATTGCTAGAATGTTCTCTTGGTAAACATTCTAATATATTTCTTTGGGCCTCTAGTGGATCGA
ATGGATGATGCAACAGAAAAACAGAGAGGATGACACCTTCGGTTCAGGTGTTGTTCAAGCTG
GGCCGTGCATGGTTCATGATAAACAAACATGTTTGCTTCTAGTCAAAATTATTGAGATTTGT
GGTGGACGAACTGGTGAAGCATCATCGATGATAAACACCATGGATGGTGCCTGGTTTATTCA
ACTTGCGTCCTCTATTTGTGACAACCTTCACCACAAGATGTTACTTTCTGAGGTAGCTCTTT
TACGCTCAAATATATGTTTGCCATATTTTCTAGTGAACTACTCCCTCCGTTCCAAAATAGAT
GACCCAACTTTGTACTAGTTAGTACAAAGTTGGGTCATCTATTTTGGAACGGAGGGAGTACA
TGGTTGATGGTGTTTTTCCCATTTCAAGAACTGAACACAATGACAGATCACTGAAGTATTTT
CACCCTTTCTTGGTTACTTGCGTGCAATGAATAACTCAAGCGTGCTCTCAAGCAAGCTCCAA
AACATTACGGATCTCATGCTAGTTTTGGCTTGTAAATTAGCTTATAATAACTTCTTTTAATT
CGGCGCTAGAATTCATCGTCTTTCTTTTGTACCTGCTCCATGCAAGCAATATTGCCATGTAC
ATACATACCAACTATGAATCAACACCTTTTTGTTAGAAACAGAGCCACACATTAGACATCTA
TCAAACTAACTATGCAATTTATTTACAGGACACCAAAAGGAACGAGGCGGCAATGAGCCACA
TGGACGAGAGAATTGAGGCAGGCATGCAGGAACTCACCCAGAATGTCCTCCAGGCGCACGGT
GATGGGACGAGCAGCGACACGAAGCAAACCTTGTTGAGCGTCGTGAGGAGCTGTTACTACGC

CGCTAACTGCCCTCCCCATGTGTTCGATGGACATGTCTCCAAGGTCATTTTCGAACATGTGT
TTTGA*AGGAAAGAAAGAAGAAATTGTCATGCAATGGTAGGAATGGGTGGAGGTGGGATCTGT*
ATGTGCCGTACATCGAAAATAATGTTGTAAGGAGTGCTAGTGAAAGAAAACAACTTGATTAA
TGCTGTAAGGAGTGCTAATTATTAAAGGTTGCACTATATTTCATTATTAAAGGAAGAGGCCA
CACAACAGTACTGGGCCTTCGCCTGTGCTCCGGCGACTAGGCCAAATTCATCAGCTCGTGCA
AACATCATCAACGGACGAAAAAAATTTAGATATTTCATATTGAATAGTCCCACCTTGCTCCA
CAAAACTGAATCTTGCCAATTTCTATATGTTCAATGTTGATGTCTGTCGTGAGCTAATTAAA
TAAGATCTCTTTTTTTTTGCGGAATAATTAAACGGGATCTCTAACATGCAAGGAAATCGTGA
GTGTATGTGGGCTGCCTGGTATTTAAACTTTGCATTGTGCATATTATGAAAGGAATGATTGT
GCATGGGCTCGAAAAAAGTGCATAATTAAAGATGAAGGGTTACGGGTGGAGTAA

Supplementary Figure S4A Genomic sequence and gene model of *LMA-1* in Chinese Spring. Start and Stop codons are highlighted in red. Intron junctions are highlighted in blue and pink respectively, intronic sequence is highlighted in grey. UTRs are in italics, and the mutation that interrupts the ORF is in red and bold font.

MQLHLSPPAWVPSGYGHGPRPRALVIKGPCRFRGKGDAALVETAGSRVALQIAQATSVSSAN
GLQTNVVRNDIQVLERTEEQCELDDCCVIPGTEFEQPLVDQVRMMLGSMSDGEINVSAYDTA
WVALVPSLDDCDSPQFPTTLRWILDNQLPDGSWGDAALFSAYDQVINTLACVVALTKWSLGP
DKCRRGLSFLEENVWRLAEEDLESMPIGFEIVFPSLLEVAKSLGIGFPYDHHALQRIYANRE
VKLKRIPMEMMHRIPTSILHSLEGMPGVDWQKILRLQSSDGSFLYSPSATACALMQTGDEKC
FEYID* IVKKFNGVPNVYPVDLFERIWAVDRLERLGISRYFKQEIKQCLDYVHRHWTDEGIS
WARNSTVIDVDDTSMAFRLLRLHGYDVSPTVFEKFEKDGEFFCFVGQSTQAVTGMYNLNRAS
QVRFPGEDLLQHAGRFSYEFLREREARGTIRDKWIIAKDLPGEVKYTLDFPWYASLPRVEAR
VYLDQYGGDNDVWIGKTLYRMPLVNNNTYLELAKRDFNRCQVQHQLEWHGLQKWFTENGLET
FGVTLRDVLRVYFLAAACIFEPSRATERLAWAKVSVLANIITKYLHSDLSGNEMMERFMQGG
IYEGNSNISWHKGGAKEDILVGAFEQLIDLLAQEALPVGEGPVYINNLLRCAWIEWMMQQKN
REDDTFGSGVVQAGPCMVHDKQTCLLLVKIIEICGGRTGEASSMINTMDGAWFIQLASSICD
NLHHKMLLSEDTKRNEAAMSHMDERIEAGMQELTQNVLQAHGDGTSSDTKQTLLSVVRSCYY
AANCPPHVFDGHVSKVIFEHVF*

Supplementary Figure S4B Deduced protein sequence for the *LMA-1* gene in Chinese Spring. The premature stop is indicated by red asterisk. Because of the use of CS as a reference, codons have been translated beyond the termination point; these amino acids are shown in grey font.
