# Supplementary material for: A Major Locus on Wheat Chromosome 7B Associated With Late-Maturity α-Amylase Encodes a Putative ent-Copalyl Diphosphate Synthase
Source: Front Plant Sci. 2021 Feb 26;12:637685. doi: 10.3389/fpls.2021.637685 (PMC7952997; doi:10.3389/fpls.2021.637685)
Supplement: Supplementary file 6 [file Presentation_5.pdf]

Chinese Spring
MQLHLSPPAWVPSGYGHGPRPRALVIKGPCRFRGKGDAALVETAGSRVALQIAQATSVSS 60
Spica
MQLHLSPPAWVPSGYGHGPRPRALV**V**KGPCRFRGKGDAALVETAGSRVALQIAQATSISS
Maringa
MQLHLSPPAWVPSGYGHGPRPRALV**V**KGPCRFRGKGDAALVETAGSRVALQIAQATSVSS

*************************:*******************************:**

Chinese Spring
ANGLQTNVVRNDIQVLERTEEQCELDDCCVIPGTEFEQPLVDQVRMMLGSMSDGEINVSA 120
Spica
ANGLQTNVVRNDIQVLERTEEQCELDDCCVIPGTEFEQPLVDQVRMMLGSMSDGEINVSA
Maringa
ANGLQTNIVRNDIQVLERTEEQCELDDCCVIPGTEFEQPLVDQVRMMLGSMSDGEINVSA

*******:**

Chinese Spring
YDTAWVALVPSLDDCDSPQFPTTLRWILDNQLPDGSWGDAALFSAYDQVINTLACVVALT 180
Spica
YDTAWVALVPSLDDCDSPQFPTTLRWILDNQLPDGSWGDAALFSAYDQVINTLACVVALT
Maringa
YDTAWVALVPSLDDCDSPQFPTTLRWILDNQLPDGS**C**GDAALFSAYDQVINTLACVVALT
****
****

Chinese Spring
KWSLGPDKCRRGLSFLEENVWRLAEEDLESMPIGFEIVFPSLLEVAKSLGIGFPYDHHAL 240
Spica
KWSLGPDKCRRGLSFLEENVWRLAEEDLESMPIGFEIVFPSLLEVAKSLGIGFPYDHHAL
Maringa
KWSLGPDKCRRGLSFLEENVWRLAEEDLESMPIGFEIVFPSLLEVAKSLGIGFPYDHHAL

****

Chinese Spring
QRIYANREVKLKRIPMEMMHRIPTSIL**H**SLEGMPGVDWQKILRLQSSDGSFLYSPSATAC 300
Spica
QRIYANREVKLKRIPMEMMHRIPTSIL**H**SLEGMPGVDWQKILRLQSSDGSFLYSPSATAC
Maringa
QRIYANREVKLKRIPMEMMHRIPTSIL**H**SLEGMPGVDWQKILRLQSSDGSFLYSPSATAC

****

Chinese Spring
ALMQTGDEKCFEYID*****IVKKFNGVP**N**VYPVDLFE**R**IWAVDRLERLGISRYFKQEIKQCLD 360
Spica
ALMQTGDEKCFEYIDRIVKKFNGVP**N**VYPVDLFE**R**IWAVDRLERLGISRYFKQEIKQCLD
Maringa
ALMQTGDEKCFEYIDRIVKKFNGVP**N**VYPVDLFE**R**IWAVDRLERLGISRYFKQEIKQCLD
****
****

Chinese Spring
YVHRHWTDEGISWARNSTVI**D**V**DD**TSMAFRLLRLHGYDVSPTVFEKFEKDGEFFCFVGQS 420
Spica
YVHRHWTDEGISWARNSTVI**D**V**DD**TSMAFRLLRLHGYDVSPTVFEKFEKDGEFFCFVGQS
Maringa
YVHRHWTDEGISWARNSTVI**D**V**DD**TSMAFRLLRLHGYDVSPTVFEKFEKDGEFFCFVGQS

```
************************************************************

Chinese Spring
TQAVTGMYNLNRASQVRFPGEDLLQHAGRFSYEFLREREARGTIRDKWIIAKDLPGEVKY 480
Spica
TQAVTGMYNLNRASQVRFPGEDLLQHAGRFSYEFLREREARGTIRDKWIIAKDLPGEVKY
Maringa
TQAVTGMYNLNRASQVRFPGEDLLQHAGRFSYEFLREREARGTIRDKWIIAKDLPGEVKY

************************************************************

Chinese Spring
TLDFPWYASLPRVEARVYLDQYGGDNDVWIGKTLYRMPLVNNNTYLELAKRDFNRCQVQH 540
Spica
TLDFPWYASLPRVEARVYLDQYGGDNDVWIGKTLYRMPLVNNNTYLELAKRDFNRCQVQH
Maringa
TLDFPWYASLPRVEARVYLDQYGGDNDVWIGKTLYRMPLVNNNTYLELAKRDFNRCQVQH

************************************************************

Chinese Spring
QLEWHGLQKWFTENGLETFGVTLRDVLRVYFLAAACIFEPSRATERLAWAKVSVLANIIT 600
Spica
QLEWHGLQKWFTENGLETFGVTLRDVLRVYFLAAACIFEPSRATERLAWAKVSVLANIIT
Maringa
QLEWHGLQKWFTENGLETFGVTLRDVLRVYFLAAACIFEPSRATERLAWAKVSVLANIIT

************************************************************

Chinese Spring
KYLHSDLSGNEMMERFMQGGIYEGNSNISWHKGGAKEDILVGAFEQLIDLLAQEALPVGE 660
Spica
KYLHSDLSGNEMMERFMQGSIYEGNSNISWHKGGAKEDILVGAFEQLIDLLAQEALPVGE
Maringa
KYLHSDLSGNEMMERFMQGGIYEGNSNISWHKGGAKEDILVGAFEQLIDLLAQEALPVGE

*******************.****************************************

Chinese Spring
GPVYINNLLRCAWIEWMMQQKNREDDTFGSGVVQAGPCMVHDKQTCLLLVKIIEICGGRT 720
Spica
GPVYINNLLRCAWIEWMMQQKNREDDTFGSGVVQAGPCMVHDKQTCLLLVKIIEICGGRT
Maringa
GPVYINNLLRCAWIEWMMQQKNREDDTFGSGVVQAGPCMVHDKQTCLLLVKIIEICGGRT

************************************************************

Chinese Spring
GEASSMINTMDGAWFIQLASSICDNLHHKMLLSEDTKRNEAAMSHMDERIEAGMQELTQN 780
Spica
GEASSMINTMDGAWFIQLASSICDNLHHKMLLSEDTKRNEAAMSHMDERIEAGMQELTQN
Maringa
GEASSMINTMDGAWFIQLASSICDNLHHKMLLSEDTKRNEAAMSHMDERIEAGMQELTQN

************************************************************

Chinese Spring      VLQAHGDGTSSDTKQTLLSVVRSCYYAANCPPHVFDGHVSKVIFEHVF
828
Spica               VLQAHGDGTSSDTKQTLLSVVRSCYYAANCPPHVFDGHVSKVIFEHVF
Maringa             VLQAHGDGTSSDTKQTLLSVVRSCYYAANCPPHVFDGHVSKVIFEHVF
```

****

Supplementary Figure S5. ClustalW alignment of the deduced protein sequences of LMA-1 for Chinese Spring, Spica and Maringa. The Chinese Spring sequence was translated beyond the Stop codon (indicated as *) for comparative purpose. Amino acid differences are highlighted in light blue. Catalytic motifs previously reported for plant CPS enzymes that are present in this sequence include: an aspartate-rich DxDD (highlighted in yellow) that acts as the catalytic (Bronsted) acid (Prisic et al. 2007) and a histidine-asparagine dyad conserved as LHS and PNV motifs (highlighted in purple) that help form the catalytic base (Lemke et al. 2019). A histidine present in many CPS with a known role in GA biosynthesis is replaced in LMA-1 by arginine (highlighted in blue) found in many CPS with a role in secondary metabolism (Mann et al. 2010).
