# Supplementary material for: A Major Locus on Wheat Chromosome 7B Associated With Late-Maturity α-Amylase Encodes a Putative ent-Copalyl Diphosphate Synthase
Source: Front Plant Sci. 2021 Feb 26;12:637685. doi: 10.3389/fpls.2021.637685 (PMC7952997; doi:10.3389/fpls.2021.637685)
Supplement: Supplementary file 7 [file Presentation_6.pdf]

```
BAH56560.1
MMQLHLLPPAWVPSGYGGHGPRPRALAVKGPCRFSGKGDATLVETAGSRVALQIAQATSV
Spica           -MQLHLSPPAWVPSGYG-
HGPRPRALVVKGPCRFRGKGDAALVETAGSRVALQIAQATSI
BAP01383.1      --------------------------------------------------
ARRTSSVSSS
BAH56559.1      -----------------
MLTFTAALRHVPVLDQPTSEPWRRLSLHLHSQRRPCGLVLISK
BAP01384.1      ------------------------------------------------
QRRRSGLVISSK
BAH56558.1      -----------------
MQVFNGNLCTASYVRQANVPWRQQSLLMLQARHRRRGGVVNVQ


BAH56560.1      SSAKVLQTNLVRNDIQILER-
TEEPCDLDDYCVIPGAEFGQPLVNQVRMMLGSMSDGE--
Spica           SSANGLQTNVVRNDIQVLER-
TEEQCELDDCCVIPGTEFEQPLVDQVRMMLGSMSDGE--
BAP01383.1
KVPARLQANPTGNDVQILERPEEEARYVDEYHVIPGTELEQPLIDQVKAMLGSMGDGE--
BAH56559.1      SP-------------
SYPEVDVGEWKVDEYRQRTDEPSETRQMIDDIRTALASLGDDETS
BAP01384.1      SP-------------
SYPEAEEGEWKVDEYKRHTDEPSKKQEMINAIRTALGSLGDDETA
BAH56558.1
TSGPAQAQDRPRNQKIDPQLAPVDVQGGSRHMAHHDPSELALMIDTIRAGMSSMGGGE--
                .                 :    :    .       ..    ::: ::
:.*:...*

BAH56560.1
ISVSAYDTAWVALVPRLDDGDSPQFPATLQWILDNQLPDGSWGDAALFSAYDRITNTLAC
Spica
INVSAYDTAWVALVPSLDDCDSPQFPTTLRWILDNQLPDGSWGDAALFSAYDQVINTLAC
BAP01383.1
ISVSAYDTAWVALVPRLDGGDGAQFAAALRWILGNQLPDGSWGDAALFSAYDRITNTLAC
BAH56559.1
MSVSAYDTALVALVKNLDGGDGPQFPSCIDWIVQNQLPDGSWGDPAFFMVQDRMISTLAC
BAP01384.1
TTASAYDTALVALVKNLDGDEKPQFPSCIDWIVQNQLPDGSWGDPAFFMVQDRMISTLAC
BAH56558.1
ITVSPYDTAFVALVGDLDGGDGPQFPSSIDWIVQNQLPDGSWGESSFFLIQDRIMNTLAC
                  ..*.**** ****  **. : .**.: : **: *********:.::*
*:: .****

BAH56560.1      VVALTKWSLGPD-
KCSRGLSFLEENMWRLAEEDLESMPIGFEIAFPSLLEVAKSLGIGFP
Spica           VVALTKWSLGPD-
KCRRGLSFLEENVWRLAEEDLESMPIGFEIVFPSLLEVAKSLGIGFP
BAP01383.1      VVALTKWSLGPE-
NCRRGLSFLEDNMWRLAEEDSESMPIGFEIAFPSLLEAARRLDVVFP
BAH56559.1
VVAVKSWNIDRDNLCDRGVLFIKENMSRLVEEEQDWMPCGFEINFPALLEKAKDLDLDIP
BAP01384.1
VVAVKSWNIDCDDLCDRGVLFIQENMSRLVEEEQDWMPCGFEINLPAILEKAKDLDLDIP
BAH56558.1      VVALTSWNLHVD-
KCEKGLKFIRENMWRLAEQEEDWTLIGFEISFPSLLCMARDLGLDIP
                ***:..*.:  :  * :*: *:.:*: **.*:: :    **** :*::*  *:
*.: :*

BAH56560.1      YDHHALQRIYANREVKLKRIPMEMMHRIPTTILHSLEGMPGV--
DWHKILRLQSSDGSFL
```

Spica   YDHHALQRIYANREVKLKRIPMEMMHRIPTSILHSLEGMPGV--
DWQKILRLQSSDGSFL
BAP01383.1   YDHHALQRIYANREVKLKKIPMEMMHSIPTTILHSLEGMPGV--
DWRKILRLQSSDGSFL
BAH56559.1
YDHPVLEEIYAKRNLKLLKIPLDVLHAIPTTLLFSVEGMVDLPLDWEKLLRLRCPDGSFH
BAP01384.1
YNHPVLEEIYAKRNLKLSKIPLDVLHASPTTLLYSVEGMVDLQLDWERLLKLRCLDGSFH
BAH56558.1   YDHPALDAIYSQRNQKLSKIPLDILHSIPTTLLHSVEGMVDL--
DWQRLLKLKCSDGSFR
                : .*: **::*: ** :**::::* **::*.*:*** .:
.::*:*:. **

BAH56560.1
YSPSATAFALKQTGDAKCFEYIDRIVKKFNGGVPNVYPVDLFEHIWVVDRLERLGISRYF
Spica   YSPSATACALMQTGDEKCFEYIDRIVKKFNG-
VPNVYPVDLFERIWAVDRLERLGISRYF
BAP01383.1
FSPAATASALMQTGDTKCFEYIDRIVKKFDGGVPNVYPVDLFEHIWAVDRLERLGISRYF
BAH56559.1
SSPAATAAALSHTGDKECHAFLDRLIQKFEGGVPCSHSMDTFEQLWVVDRLMRLGISRHF
BAP01384.1
SSPAATAAALSYTGDKECLAFLDRLVKKFRGGVPCMYSLDIFEQLWVVDRLMRLGISRHF
BAH56558.1
TSPAATARALKETGDLKCLKYIDEIVKKFDGGAPCVYPVDLYERLWAVDRLTRLGISRHF
                 :* ** *** :* ::*.:::** * .* :.:* :*::*.****
******:*

BAH56560.1   KQEIKQCLDYVHRHWTEDG---
ICWARNSAVRDVDDTAMAFRLLRLHGYTVSPSVFENFE
Spica   KQEIKQCLDYVHRHWTDEG---
ISWARNSTVIDVDDTSMAFRLLRLHGYDVSPTVFEKFE
BAP01383.1   KQEIKQCLDYVHRHWTEGG---
ICWARNSAVVDVDDTAMAFRLLRLHRYNVSPRVFENFE
BAH56559.1   TSEIQQCLEFIYRRWTQKG---
LAHNMHCPIPDIDDTAMGFRLLRQHGYDVTPSVFKHFE
BAP01384.1   TSEIEQCLDCIYRRWTRKG---
LAHNVHCPTPDIDDTAMGFRLLRQHGYDVTPCVFNHFE
BAH56558.1
TSEIKECLDFTYRHWTQVEDDGLSHAGSCSAADIDDTAMGFRLLRLNGYHVNPCALKKFE
                ..**::**: :*:**   :.   .. *:***:*.***** : * *.*
.:::**

BAH56560.1   --KDGEFFCFAGQS-TQAVTGMYNLNRASQLRFPGE--
DVLQRAGRFSYAFLREREAQGT
Spica   --KDGEFFCFVGQS-TQAVTGMYNLNRASQVRFPGE--
DLLQHAGRFSYEFLREREARGT
BAP01383.1   --KDGDFFCFVGQS-TQAVTGMYNLNRASQVRFPGE--
DVLQRAGRFSYEFLREREAQDT
BAH56559.1   --KDGKFVCFPMETNHASVTPMHNTYRASQFMFPGD-
DDVLARAGRYCRAFLQERQSSNK
BAP01384.1   DKKDGKFFCFPLETNDASVTPMYNTYRASQFMFPGD-
DDVLARAGRYCRAFLHERQASNK
BAH56558.1   --KDGEFFCFPRQS-
SQSVTAIYNTYRATQVAFPGEKDDVLRRAEQFGRAFLQERRASNK
                  ***.*.** ::   :** ::* **:*. ***: *:* :* ::
:.: ..

BAH56560.1
IRDKWIIAKDLPGEVKYTLDFPWYASLPRVEARAYLDQYGGENDVWIGKTLYRMPLVNNN

Spica
IRDKWIIAKDLPGEVKYTLDFPWYASLPRVEARVYLDQYGGDNDVWIGKTLYRMPLVNNN
BAP01383.1
IRDKWIISKDLPGEVQYTLDFPWYASLPRVEARIYLDQYGGDDDVWIGKTLYRMPLVNNN
BAH56559.1
LYDKWIITKDLPGEVGYTLNFPWKSSLPRIETRMYLDQYGGNNDVWIAKVLYRMNLVSND
BAP01384.1
LYDKWIITKDLPGEVEYALNFPWEASLPRIETRMYLDQYGGNTDVWIANVLYRMNLVSND
BAH56558.1
LNDKWVIPKDLPGEVEYALDFPWKASLPRVETRFYLEQYGGGDDVWIGKVLYRMPLVNND
: ***:*.******* *:*:*** :****:*:* **:****
****.:.**** **.*:

BAH56560.1
TYLELAKRDFNRCQVQHQLEWHGLQKWFIENGLETFGVALRDVLRAYFLAAACIFEPSRA
Spica
TYLELAKRDFNRCQVQHQLEWHGLQKWFTENGLETFGVTLRDVLRVYFLAAACIFEPSRA
BAP01383.1
TYLELAKRDFNRCQVQHQLEWHGLQKWFTENGLEAFGVAPRDVLRAYFLAAACIFEPSRA
BAH56559.1
LYLKMAKADFTEYQRLSRIEWNGLRKWYFRNHLQRYGATPKSALKAYFLASANIFEPGRA
BAP01384.1
LYLEMAKADFREYQRLFRLEWNGLRKWYLRNHLQKYGGTPKSALTAYFLASANIFESGRA
BAH56558.1
LYLEAAKADFTNFQRMCRLEWHGLKRWYERNNLEMYGVSANSALRAYFLAAANIFEPTRA
: ** ** . * :::**::*: .* *: :* : ...* .****:*
***. **

BAH56560.1
TERLAWAKVSVLANIITKYLHSDLSGNEMMERFMHGGLHEGNSTISWRKGDAKGDILLGA
Spica
TERLAWAKVSVLANIITKYLHSDLSGNEMMERFMQGSIYEGNSNISWHKGGAKEDILVGA
BAP01383.1
TERLAWSRASVMANIISKYLRSDLSGNKMVERFMHGGLYEGNNDVSWLKGDAKVEILVGA
BAH56559.1 AERLAWARMAVLAEAVTTHFRHIGGPCYSTENLEELIDLVSFD----------
----
BAP01384.1 AERLAWARTWVLAEAVTSHFRHTGGTKDSTKNLEELIDIVSFD----------
----
BAH56558.1
AERLAWARTAMLAEAVSSYFQRNGCAPELRERLSAILTPGHSHNLARGAMDSVENSIP--
:*****:: ::*: ::.::: :.:

BAH56560.1
LQQFIDLLAQETLPVGGGPVYINNLLRCAWIEWMMQLINREDDTYDSSVIQAGSCMVHDK
Spica
FEQLIDLLAQEALPVGEGPVYINNLLRCAWIEWMMQQKNREDDTFGSGVVQAGPCMVHDK
BAP01383.1
LEKLIDLLAQKALHVGEEPMHINNLLRCVWIEWMMQEINRDDGTNGMSVIEAGSCMVHDK
BAH56559.1 -------------------DVSGG-LREAWKQWLMAWTAKE------------
SHGSVDG
BAP01384.1 -------------------DDSSGSLRDAWKQWLMAWTAKE------------
SHGSIEG
BAH56558.1 ------HVVNELIMDLSKFDNAADTLREAWKDWFTTWTAKE------------
SSGPCEW
. ** .* :*: ::
. :

BAH56560.1
QTCLLSSQIVEICGGRTAEASSMINSMDGACFIQLASSICDNLHHKMLLSQDTKTNETAM
Spica
QTCLLLVKIIEICGGRTGEASSMINTMDGAWFIQLASSICDNLHHKMLLSEDTKRNEAAM

```
BAP01383.1
QTSLLLVKIIEICAGRIGEASSMVNSKDNTWFIQLASSICDSLHHRMLLSQDTEENKAVT
BAH56559.1
DTALLFVRTIEICSGRIVSSEQKLNLWDYSQLEQLTSSICHKLATIGLSQNEA--SMENT
BAP01384.1
DTALLLVRTVEITSGRHVSAEQKLNLWEYSQLEQLTSSICRKLATRVLAQNGE--NMENT
BAH56558.1
STALLLVRTVEVCSGRHGSTQQQLNISEYTQLEGLTSSICRKLSSRVLSQGGQ--KMENI
                .*.**  : :*: .**  .:.. :*  : : :  *:**** .*    * .
.

BAH56560.1
SHMNEKIEAGMQELTQKFLQTHDDGTSSETKRTLLSVVRSCYYAANCPHHVFDRHVSKVI
Spica
SHMDERIEAGMQELTQNVLQAHGDGTSSDTKQTLLSVVRSCYYAANCPPHVFDGHVSKVI
BAP01383.1
SHMDKKIEVDMQELAQNILQTYDDISSNNMKQTFWSVVKSCYYVANCPSYILDRHVSKVI
BAH56559.1      EDLHQQVDLEMQELSWRVHQGCHGIN-
RETRQTFLNVVKSFYYSAHCSPETVDSHIAKVI
BAP01384.1      EDLDQQVDIEMQELSWRIHQGCHGIN-
IDTRQTFLHVVKSFYYSAHCSAETVDSHIAKVV
BAH56558.1      EDIDRQVDLDMRELTQHVLQSCNGVS-
GLTRQTFMHVVKSFNYVTLCPHDTIDCHISKVL
                ..:..:::  *:**: .. *   . .    ::*:  **:*  * : *.   .*
*::**:

BAH56560.1      FEHVF
Spica           FEHVF
BAP01383.1      FEHVF
BAH56559.1      FQDVI
BAP01384.1      FQDVI
BAH56558.1      FEDVI
                *:.*:
```

Supplementary Figure S6. ClustalW alignment of Spica *LMA-1* with previously reported ent-copalyl diphosphate synthases (Wu et al 2012). BAH56558.1 corresponds to *TaCPS1*, BAH56559.1 = *TaCPS2*, BAH56560.1 = *TaCPS3*, BAP01383.1 = *TaCPS4*, BAP01384.1 = *TaCPS5*.
