# Supplementary material for: A Major Locus on Wheat Chromosome 7B Associated With Late-Maturity α-Amylase Encodes a Putative ent-Copalyl Diphosphate Synthase
Source: Front Plant Sci. 2021 Feb 26;12:637685. doi: 10.3389/fpls.2021.637685 (PMC7952997; doi:10.3389/fpls.2021.637685)
Supplement: Supplementary file 8 [file Presentation_7.pptx]

## Slide 1
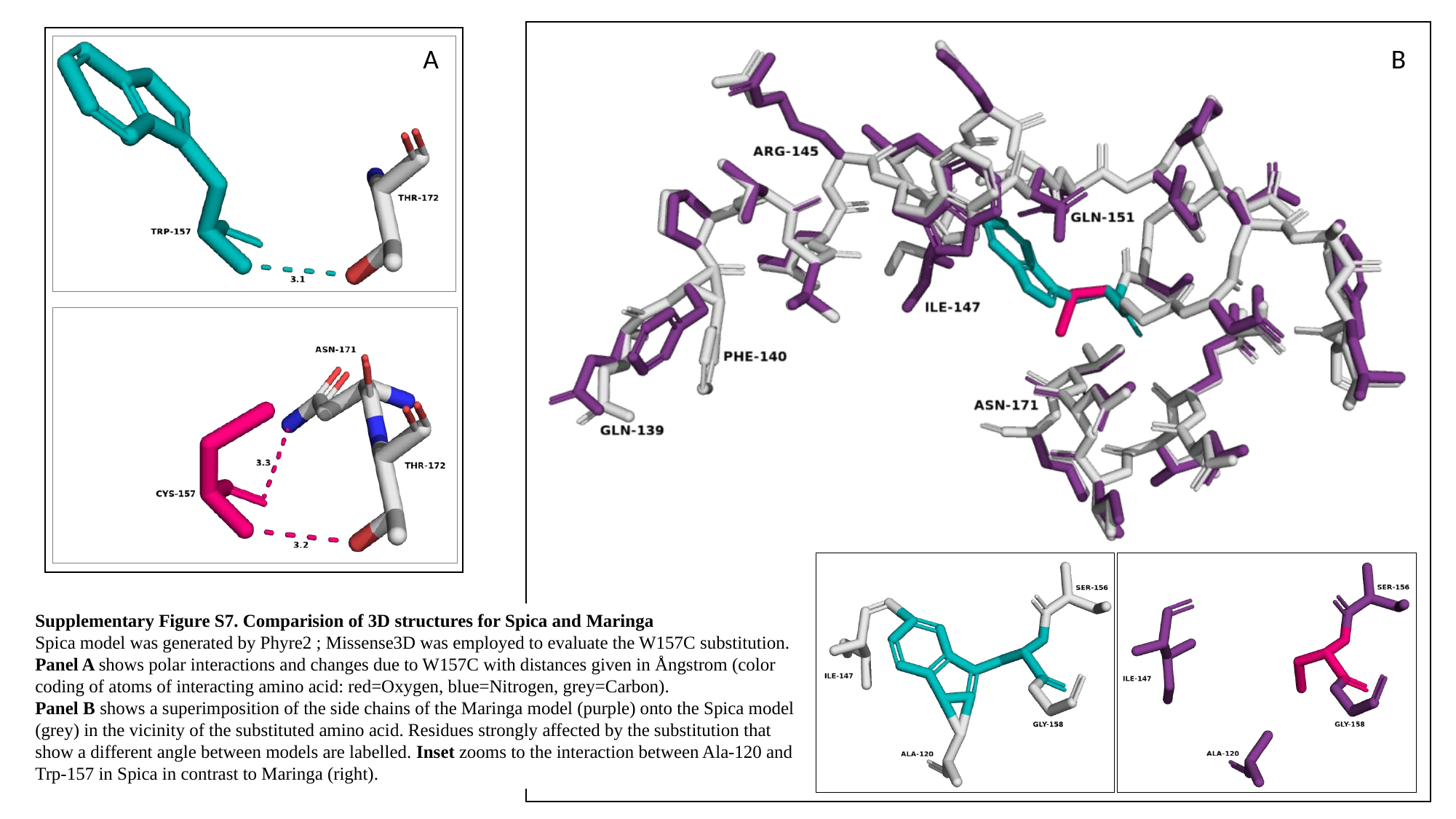

A
B
Supplementary Figure S7. Comparision of 3D structures for Spica and Maringa
Spica model was generated by Phyre2 ; Missense3D was employed to evaluate the W157C substitution.
Panel A shows polar interactions and changes due to W157C with distances given in Ångstrom (color coding of atoms of interacting amino acid: red=Oxygen, blue=Nitrogen, grey=Carbon).
Panel B shows a superimposition of the side chains of the Maringa model (purple) onto the Spica model (grey) in the vicinity of the substituted amino acid. Residues strongly affected by the substitution that show a different angle between models are labelled. Inset zooms to the interaction between Ala-120 and Trp-157 in Spica in contrast to Maringa (right).
