# Supplementary material for: A Major Locus on Wheat Chromosome 7B Associated With Late-Maturity α-Amylase Encodes a Putative ent-Copalyl Diphosphate Synthase
Source: Front Plant Sci. 2021 Feb 26;12:637685. doi: 10.3389/fpls.2021.637685 (PMC7952997; doi:10.3389/fpls.2021.637685)
Supplement: Supplementary file 9 [file Presentation_8.pptx]

## Slide 1
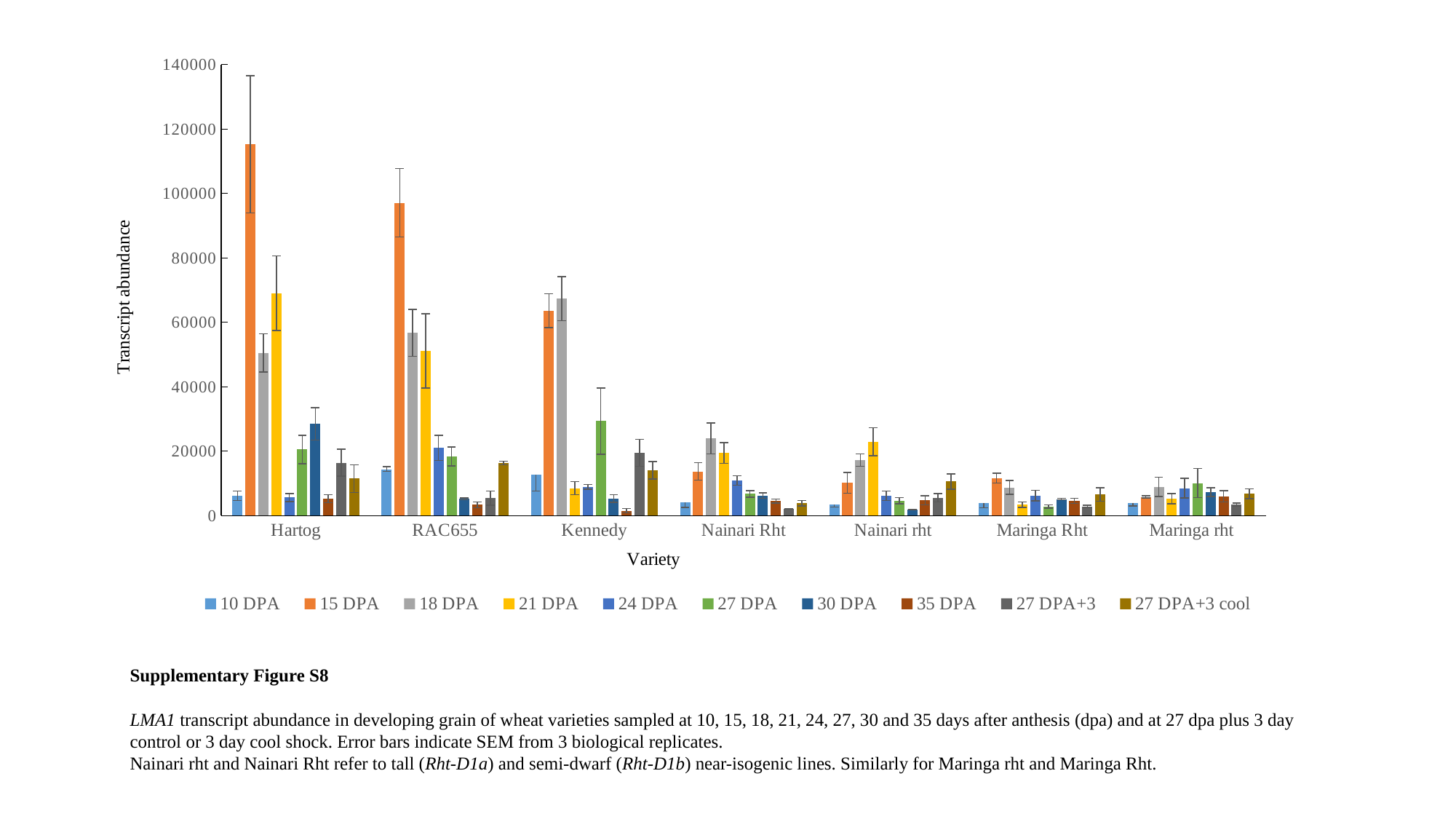

### Chart
| Category | 10 DPA | 15 DPA | 18 DPA | 21 DPA | 24 DPA | 27 DPA | 30 DPA | 35 DPA | 27 DPA+3 | 27 DPA+3 cool |
|---|---|---|---|---|---|---|---|---|---|---|
| Hartog | 6170.924996970854 | 115222.71759808953 | 50473.40596839353 | 69006.02994356779 | 5615.614016635027 | 20531.903969214483 | 28428.267382339294 | 5306.939225878169 | 16443.10874801052 | 11515.876128728296 |
| RAC655 | 14192.410356478162 | 97081.3829979266 | 56699.31997326558 | 51142.67997287243 | 21056.45992252686 | 18339.016629256443 | 5246.085299625087 | 3389.5772631381756 | 5456.629460421062 | 16332.788586151199 |
| Kennedy | 12777.067122933871 | 63587.278244696965 | 67311.16340154933 | 8545.323000590852 | 8907.929397345728 | 29331.601920280315 | 5318.349432592867 | 1542.2460154719847 | 19478.293636282848 | 14054.49084947352 |
| Nainari Rht | 4221.202637855848 | 13697.272103470677 | 23940.57942043174 | 19449.19657141054 | 10924.014633625398 | 6751.2967997931 | 6200.106867893289 | 4523.607125838626 | 2135.2499176864753 | 3836.1998240395237 |
| Nainari rht | 3457.134659820443 | 10191.065653772299 | 17262.328909572934 | 22971.2791076635 | 6212.028054198386 | 4618.004899034211 | 1882.4116826688908 | 4785.339742165585 | 5566.526731563369 | 10623.513529638441 |
| Maringa Rht | 3824.8272469293884 | 11635.64547425166 | 8766.04246322486 | 3428.905046193133 | 6236.5430728301735 | 2831.9571218213314 | 5149.367171215353 | 4608.039871745431 | 2838.3142200846373 | 6575.991013781645 |
| Maringa rht | 4027.594084303412 | 5824.822143540888 | 8917.93667683915 | 5280.025652782365 | 8542.907706898428 | 10120.426516346935 | 7329.159844948749 | 5973.176986612867 | 3388.1681646386664 | 6846.261141268253 |Supplementary Figure S8
LMA1 transcript abundance in developing grain of wheat varieties sampled at 10, 15, 18, 21, 24, 27, 30 and 35 days after anthesis (dpa) and at 27 dpa plus 3 day control or 3 day cool shock. Error bars indicate SEM from 3 biological replicates.
Nainari rht and Nainari Rht refer to tall (Rht-D1a) and semi-dwarf (Rht-D1b) near-isogenic lines. Similarly for Maringa rht and Maringa Rht.
