# Supplementary material for: A Major Locus on Wheat Chromosome 7B Associated With Late-Maturity α-Amylase Encodes a Putative ent-Copalyl Diphosphate Synthase
Source: Front Plant Sci. 2021 Feb 26;12:637685. doi: 10.3389/fpls.2021.637685 (PMC7952997; doi:10.3389/fpls.2021.637685)
Supplement: Supplementary file 10 [file Presentation_9.pptx]

## Slide 1
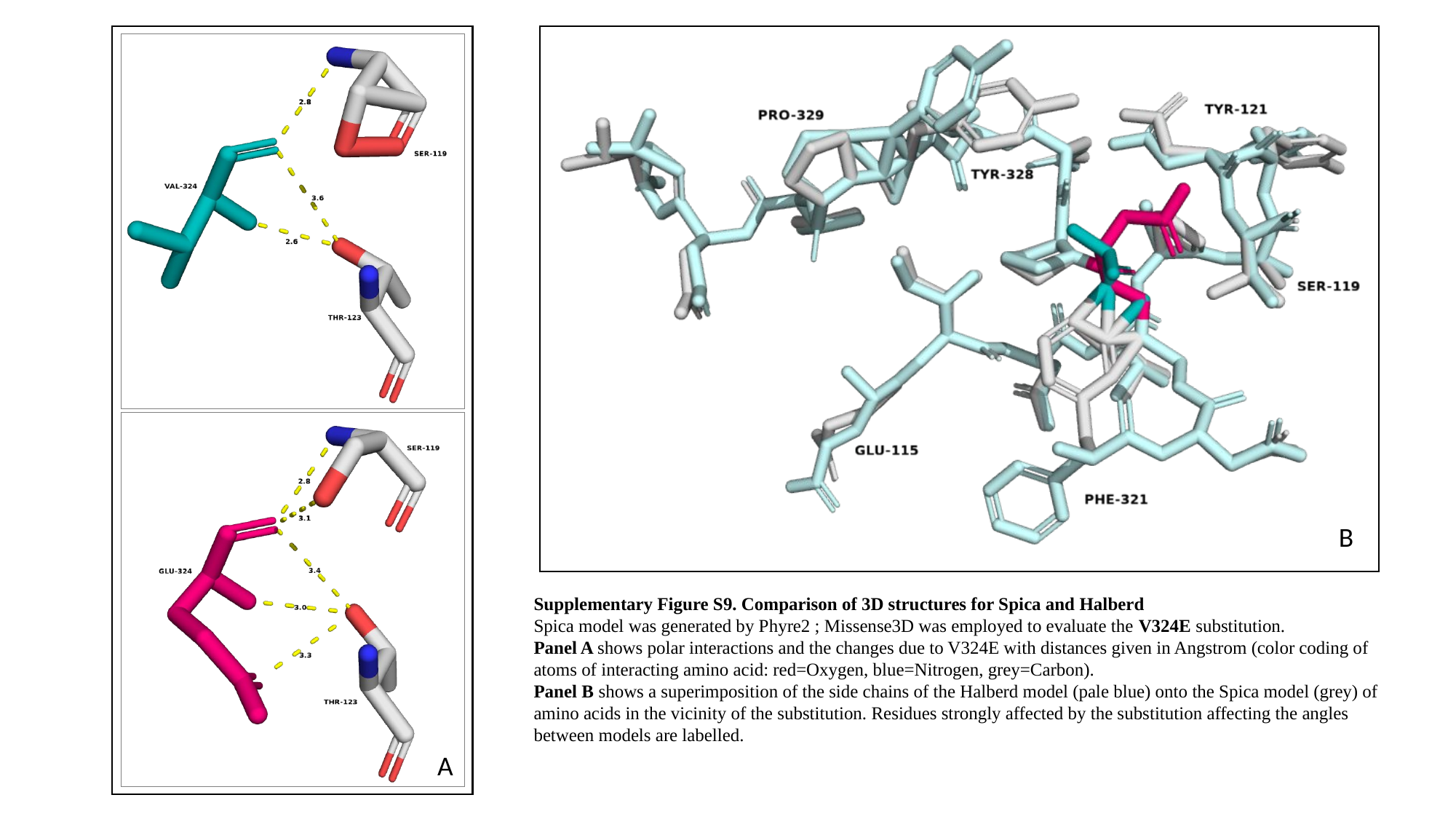

B
Supplementary Figure S9. Comparison of 3D structures for Spica and Halberd
Spica model was generated by Phyre2 ; Missense3D was employed to evaluate the V324E substitution.
Panel A shows polar interactions and the changes due to V324E with distances given in Angstrom (color coding of atoms of interacting amino acid: red=Oxygen, blue=Nitrogen, grey=Carbon).
Panel B shows a superimposition of the side chains of the Halberd model (pale blue) onto the Spica model (grey) of amino acids in the vicinity of the substitution. Residues strongly affected by the substitution affecting the angles between models are labelled.
A
