# Supplementary material for: A Major Locus on Wheat Chromosome 7B Associated With Late-Maturity α-Amylase Encodes a Putative ent-Copalyl Diphosphate Synthase
Source: Front Plant Sci. 2021 Feb 26;12:637685. doi: 10.3389/fpls.2021.637685 (PMC7952997; doi:10.3389/fpls.2021.637685)
Supplement: Supplementary file 11 [file Presentation_10.pptx]

## Slide 1
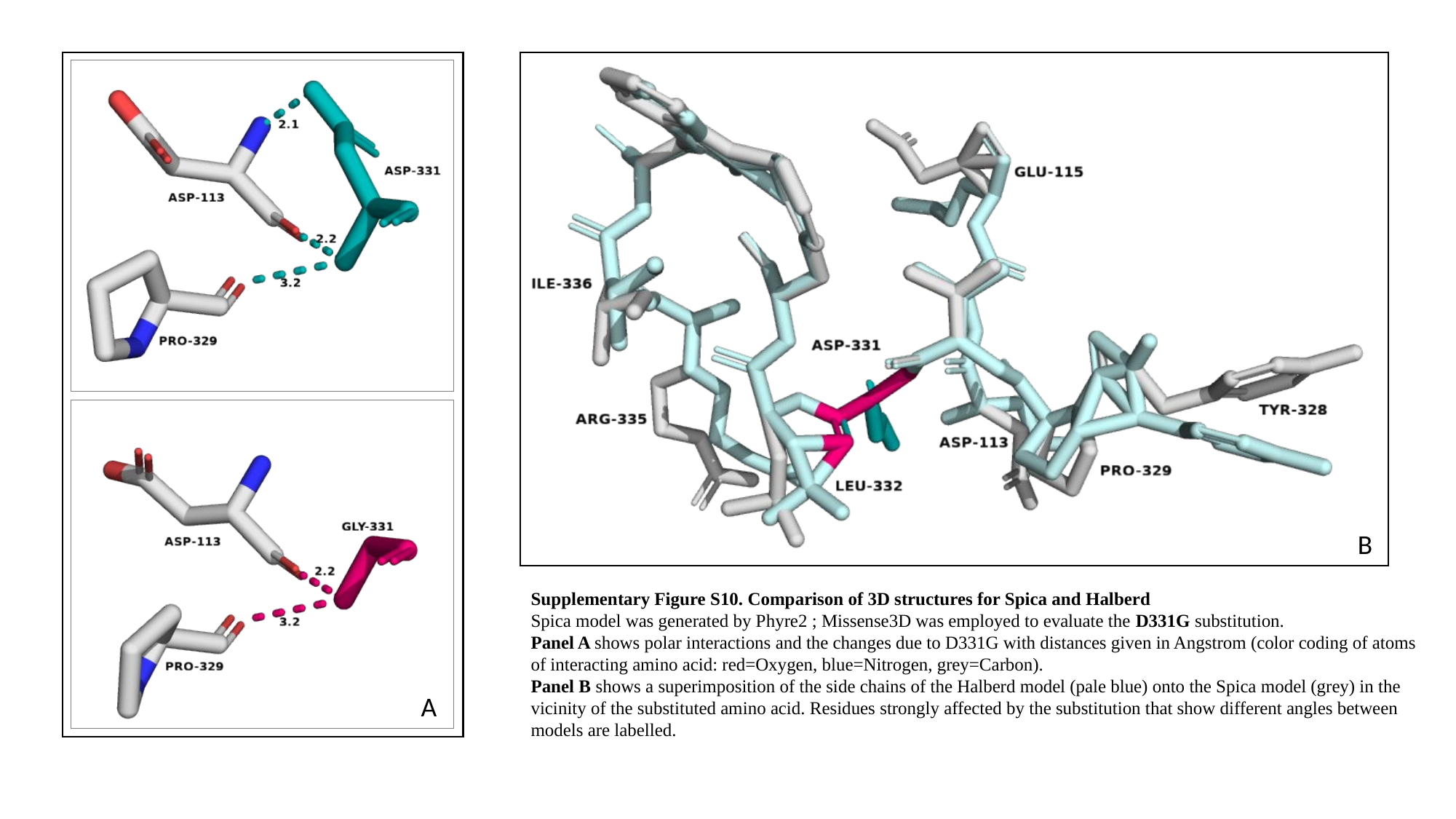

B
Supplementary Figure S10. Comparison of 3D structures for Spica and Halberd
Spica model was generated by Phyre2 ; Missense3D was employed to evaluate the D331G substitution.
Panel A shows polar interactions and the changes due to D331G with distances given in Angstrom (color coding of atoms of interacting amino acid: red=Oxygen, blue=Nitrogen, grey=Carbon).
Panel B shows a superimposition of the side chains of the Halberd model (pale blue) onto the Spica model (grey) in the vicinity of the substituted amino acid. Residues strongly affected by the substitution that show different angles between models are labelled.
A
