# Supplementary material for: A Major Locus on Wheat Chromosome 7B Associated With Late-Maturity α-Amylase Encodes a Putative ent-Copalyl Diphosphate Synthase
Source: Front Plant Sci. 2021 Feb 26;12:637685. doi: 10.3389/fpls.2021.637685 (PMC7952997; doi:10.3389/fpls.2021.637685)
Supplement: Supplementary file 12 [file Presentation_11.pptx]

## Slide 1
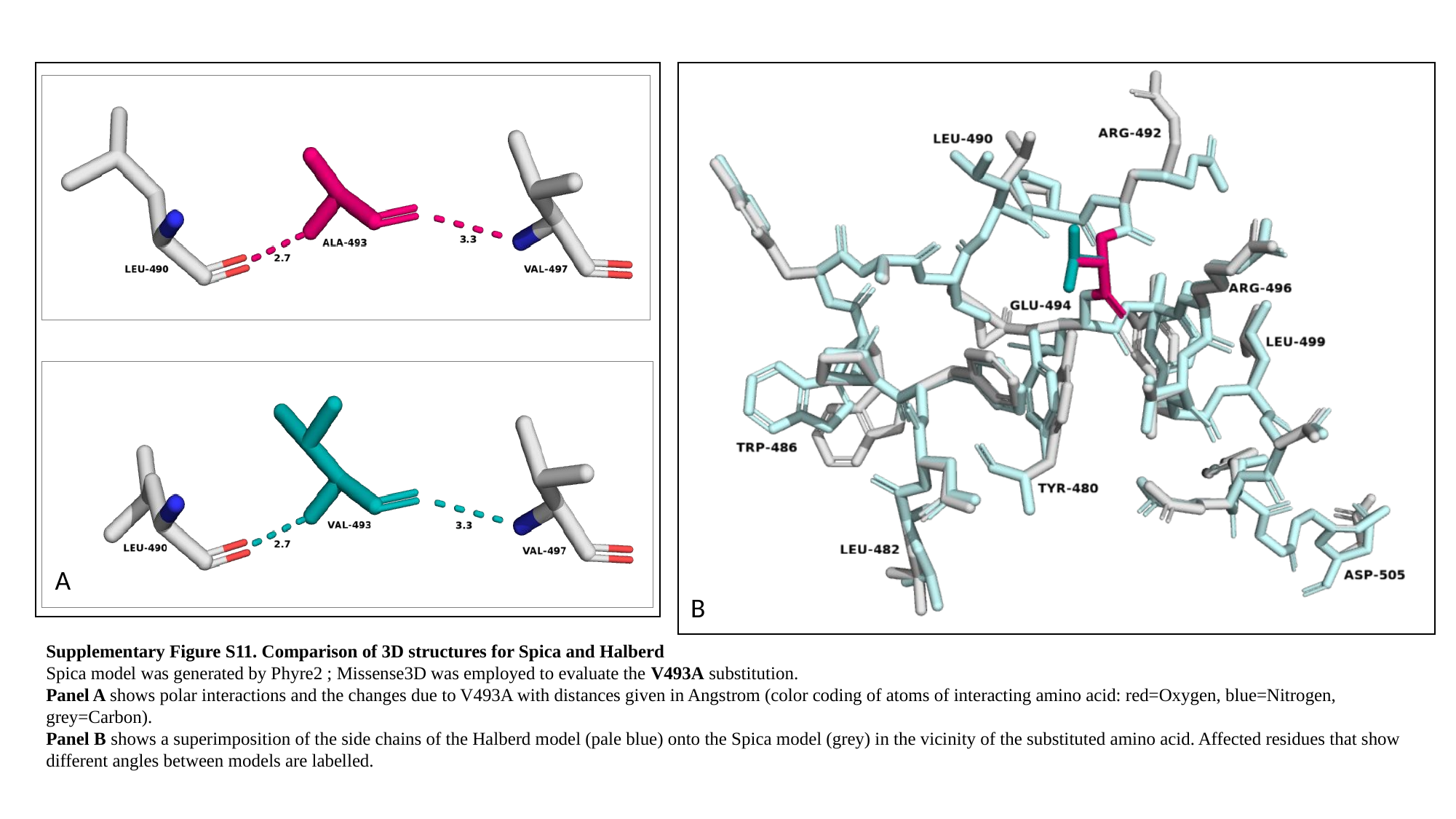

A
B
Supplementary Figure S11. Comparison of 3D structures for Spica and Halberd
Spica model was generated by Phyre2 ; Missense3D was employed to evaluate the V493A substitution.
Panel A shows polar interactions and the changes due to V493A with distances given in Angstrom (color coding of atoms of interacting amino acid: red=Oxygen, blue=Nitrogen, grey=Carbon).
Panel B shows a superimposition of the side chains of the Halberd model (pale blue) onto the Spica model (grey) in the vicinity of the substituted amino acid. Affected residues that show different angles between models are labelled.
