# Supplementary material for: A Major Locus on Wheat Chromosome 7B Associated With Late-Maturity α-Amylase Encodes a Putative ent-Copalyl Diphosphate Synthase
Source: Front Plant Sci. 2021 Feb 26;12:637685. doi: 10.3389/fpls.2021.637685 (PMC7952997; doi:10.3389/fpls.2021.637685)
Supplement: Supplementary file 13 [file Presentation_12.pptx]

## Slide 1
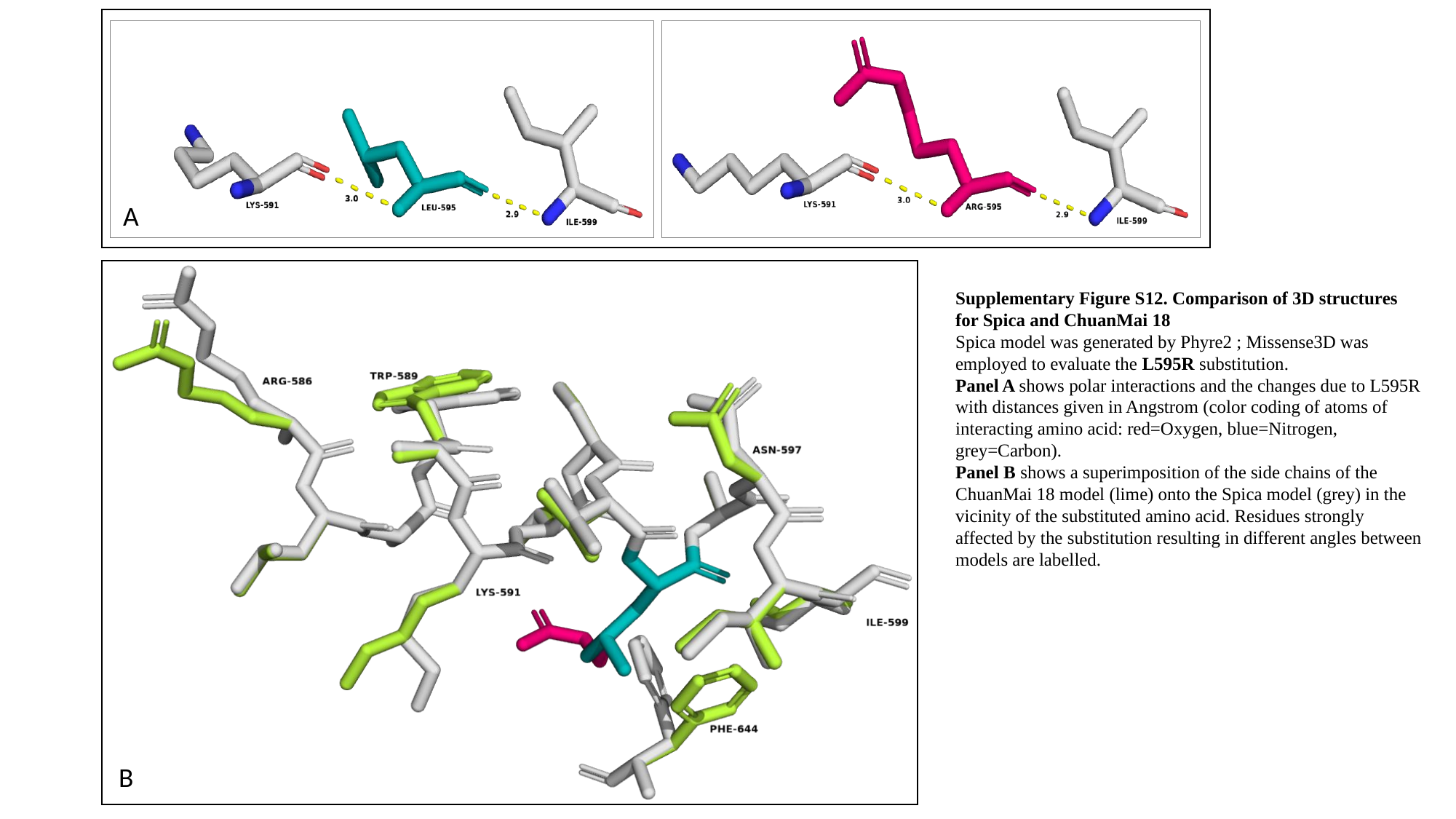

A
B
Supplementary Figure S12. Comparison of 3D structures for Spica and ChuanMai 18
Spica model was generated by Phyre2 ; Missense3D was employed to evaluate the L595R substitution.
Panel A shows polar interactions and the changes due to L595R with distances given in Angstrom (color coding of atoms of interacting amino acid: red=Oxygen, blue=Nitrogen, grey=Carbon).
Panel B shows a superimposition of the side chains of the ChuanMai 18 model (lime) onto the Spica model (grey) in the vicinity of the substituted amino acid. Residues strongly affected by the substitution resulting in different angles between models are labelled.
